# *In vivo* Modeling Implicates *APOL1* in Nephropathy: Evidence for Dominant Negative Effects and Epistasis under Anemic Stress

**DOI:** 10.1371/journal.pgen.1005349

**Published:** 2015-07-06

**Authors:** Blair R. Anderson, David N. Howell, Karen Soldano, Melanie E. Garrett, Nicholas Katsanis, Marilyn J. Telen, Erica E. Davis, Allison E. Ashley-Koch

**Affiliations:** 1 Center for Human Disease Modeling, Duke University Medical Center, Durham, North Carolina, United States of America; 2 Department of Pathology, Division of Pathology Clinical Services, Duke University, Durham, North Carolina, United States of America; 3 Department of Medicine, Division of Hematology, Duke University Medical Center, Durham, North Carolina, United States of America; Stanford University School of Medicine, UNITED STATES

## Abstract

African Americans have a disproportionate risk for developing nephropathy. This disparity has been attributed to coding variants (G1 and G2) in apolipoprotein L1 (*APOL1*); however, there is little functional evidence supporting the role of this protein in renal function. Here, we combined genetics and *in vivo* modeling to examine the role of apol1 in glomerular development and pronephric filtration and to test the pathogenic potential of *APOL1* G1 and G2. Translational suppression or CRISPR/Cas9 genome editing of *apol1* in zebrafish embryos results in podocyte loss and glomerular filtration defects. Complementation of *apol1* morphants with wild-type human *APOL1* mRNA rescues these defects. However, the *APOL1* G1 risk allele does not ameliorate defects caused by *apol1* suppression and the pathogenicity is conferred by the *cis* effect of both individual variants of the G1 risk haplotype (I384M/S342G). *In vivo* complementation studies of the G2 risk allele also indicate that the variant is deleterious to protein function. Moreover, *APOL1* G2, but not G1, expression alone promotes developmental kidney defects, suggesting a possible dominant-negative effect of the altered protein. In sickle cell disease (SCD) patients, we reported previously a genetic interaction between *APOL1* and *MYH9*. Testing this interaction *in vivo* by co-suppressing both transcripts yielded no additive effects. However, upon genetic or chemical induction of anemia, we observed a significantly exacerbated nephropathy phenotype. Furthermore, concordant with the genetic interaction observed in SCD patients, *APOL1* G2 reduces myh9 expression *in vivo*, suggesting a possible interaction between the altered APOL1 and *myh9*. Our data indicate a critical role for *APOL1* in renal function that is compromised by nephropathy-risk encoding variants. Moreover, our interaction studies indicate that the *MYH9* locus is also relevant to the phenotype in a stressed microenvironment and suggest that consideration of the context-dependent functions of both proteins will be required to develop therapeutic paradigms.

## Author Summary

African Americans have a disproportionate risk for developing chronic kidney disease compared to European Americans. Previous studies have identified a region on chromosome 22 containing two genes, *MYH9* and *APOL1*, which likely accounts for nearly all of this difference. Previous reports provided strong statistical evidence implicating *APOL1* as the major contributor to nephropathy risk in African Americans, driven by two coding variants, termed G1 and G2. However, other groups still report statistical evidence for *MYH9* association in kidney disease, and animal models have demonstrated biological relevance for *MYH9* function in the kidney. Here, we show that suppressing *apol1* in zebrafish embryos results in perturbed kidney function. Importantly, using this *in vivo* assay, we show that the G1 variant appears to cause a loss of APOL1 function, while the G2 variant results in an altered protein that may be acting antagonistically in the presence of normal APOL1. We also report a genetic interaction between *apol1* and *myh9* under anemic stress, which is consistent with our previous findings in sickle cell disease (SCD) nephropathy patients. Finally, we provide functional evidence *in vivo* that the G2-altered APOL1 may be interacting with *MYH9* to confer nephropathy risk.

## Introduction

Chronic kidney disease (CKD) is an acute public health problem world-wide. Within the United States alone, it affects up to 14% of the adult population and is associated with both high costs and poor clinical outcomes[1]. Compared with European Americans, African Americans have a disproportionate risk for several forms of CKD, including human immunodeficiency virus (HIV)-associated nephropathy, focal segmental glomerulosclerosis (FSGS), hypertension-attributed CKD, and sickle cell disease nephropathy (SCDN), all of which contribute to a four-fold increased risk of the most severe stage of CKD, end-stage renal disease (ESRD)[1–5]. A genomic region on chromosome 22q12 likely accounts for almost all of this racial disparity. This region contains two genes, non-muscle myosin heavy chain IIA (*MYH9*; Entrez, 4627) and apolipoprotein L1 (*APOL1*; Entrez, 8542), both of which have been associated with increased risk among African American patients with nondiabetic nephropathy[5–11]. Initial admixture mapping and subsequent fine mapping studies focused on *MYH9*[8, 9, 11]. However, due to the inability to identify variants in *MYH9* that alter protein sequence, the major source of genetic association has been attributed to *APOL1*, located 14 kb downstream of *MYH9*[6]. Two *APOL1* alleles, G1 (encoding p.S342G and p.I384M in *cis*) and G2 (encoding p.N388del:Y389del), comprise one of the strongest genetic signals ever reported in complex human disease (odds ratios ranging from 10.5 to 16.9)[6, 7]. Additionally, these alleles alter the protein to confer resistance to *Trypanosoma brucei rhodesiense*, offering a potential evolutionary explanation for the increased occurrence observed among individuals of African ancestry[6].

Despite these genetic findings and the association of this locus with increased risk of multiple forms of CKD, there is a dearth of functional data to inform directly whether *MYH9* or *APOL1* is the driver of this genetic association. In mice, homozygous *Myh9* knockouts die at an early embryonic stage[12], and heterozygotes appear viable without any detected abnormalities[13]. However, subsequent studies have demonstrated that knock-in mutants display renal glomerulosclerosis, while podocyte-specific deletion of *Myh9* may predispose mice to glomerulopathy[14–16]. In zebrafish, *myh9* is required for the normal development of the glomerulus; morpholino (MO)-induced *myh9* suppression results in non-uniform podocyte foot processes and glomerular basement membrane thickening[17]. In contrast, the possible relevance of APOL1 to CKD is derived primarily from *in vitro* work: cellular localization studies of *APOL1* in nondiabetic kidney disease patient biopsies suggest an implication in arteriopathy[18, 19], while overexpression of *APOL1* and its risk alleles enhance podocyte necrosis *in vitro* [20].

Nephropathy is a major contributor to early mortality in patients with sickle cell disease (SCD)[21, 22]. SCDN is a clinically well-characterized pathology that includes glomerular hypertrophy, hyposthenuria, tubular dysfunction, proteinuria, and overall progressive renal failure[23]. We reported previously an association of both *MYH9* and *APOL1* variants as independent risk factors for proteinuria in a SCD study population[5]. Additionally, when glomerular filtration rate (GFR) in SCD patients was modeled as a function of the previously reported *MYH9* risk haplotype and the *APOL1* recessive model, we observed a significant interaction between the two genes, suggesting that *APOL1* and *MYH9* may act together to induce SCDN[5]. However, as with other forms of CKD, well-characterized *in vivo* model systems are needed to understand both the individual effects of *APOL1* relevant to disease, and also the potential interaction of *APOL1* with *MYH9* in the context of anemic stress as observed in SCD.

Here, we used zebrafish as an *in vivo* model to study the consequences of gene perturbation and potential synergistic effects of *APOL1* and *MYH9* in kidney disease. Although the zebrafish pronephros is a simplified kidney, the structure and function of the larval glomerulus is similar to that of humans and represents a tractable model in which to study *apol1* (RefSeq: NM_001030138) and *myh9* (RefSeq: NM_001098177.2)[24, 25]. In this report, we provide insight into the role of *apol1* in glomerular development and pronephric filtration in zebrafish embryos, as well as the effects of *APOL1* G1 and G2 allelic expression. Moreover, we provide functional evidence for an interaction between *myh9* and *apol1* under anemic stress conditions. Overall, these data implicate both *MYH9* and *APOL1* as significant biological contributors to non-diabetic nephropathy and intimate context-dependent roles in disease pathology.

## Results

### Knockdown of zebrafish *apol1* results in pericardial edema, compromised glomerular filtration, and disruption of the glomerular ultrastructure

**Fig 1:**
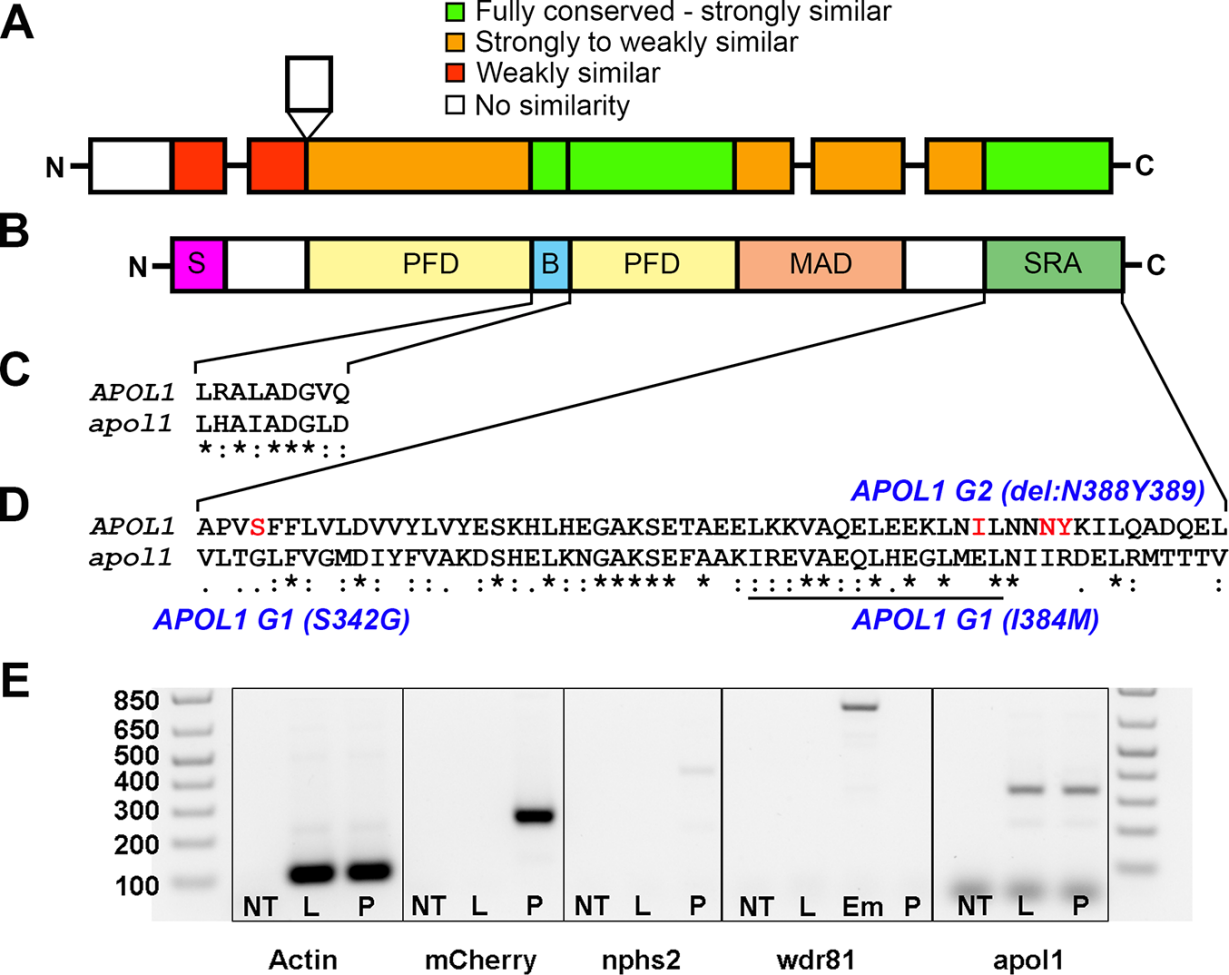
Comparison of APOL1 human and zebrafish protein sequences and relevance to the zebrafish kidney. Protein domain schematic of (A) zebrafish APOL1 and (B) human APOL1 is shown, with zebrafish domains (NP_001025309) aligned to the human protein (NP_001130012) and coded based on summarized consensus scores (Gonnet PAM 250 matrix, Clustal Omega, Cambridge, UK; *S*, secretory domain, *PFD*, pore-forming domain, *B*, BH3 domain, *MAD*, membrane-addressing domain, *SRA*, serum resistance-associated binding domain). Prominent regions of the human and zebrafish alignments are expanded, including the (C) BH3 domain and (D) SRA binding domain, and consensus symbols are displayed (* (asterisk), fully conserved;: (colon), >0.5 in the Gonnet PAM 250 matrix;. (period), = <0.5 in the Gonnet PAM 250 matrix). The leucine zipper domain (codons 365–392 in *APOL1*, underline), and the location of the G1 and G2 risk alleles in CKD in African Americans (S342G/I384M and ΔN388Y389) are highlighted in red. (E) Podocytes from adult glomeruli of *pod*::NTR-mCherry zebrafish were flow-sorted and evaluated for *apol1* RNA expression through RT-PCR. *apol1* is expressed in fluorescence-activated cell sorted (FACS) podocytes and the adult liver. FACS podocytes also express zebrafish *podocin* (*nphs2)* but a purkinje-cell marker, *wdr81*[29], was undetectable. NT = non-template reverse transcription control; L = dissected adult liver cells from *pod*::NTR-mCherry zebrafish; P = fluorescence-activated cell sorted podocytes from dissected glomeruli of *pod*::NTR-mCherry zebrafish; Em = 5 dpf whole-zebrafish embryo cDNA.

The apolipoprotein L family of genes evolved rapidly in humans and some non-human primates[26, 27]. However, using BLAST and reciprocal BLAST searches against the *D*. *rerio* and *H*. *sapiens* genomes, we identified a single *D*. *rerio* locus encoding a protein of unknown function (chr2:37,674,122–37,676,731 Zv9; NCBI Ref: NP_001025309.1; 38% identity, 46% similarity on the amino acid level) as a possible unique functional ancestral ortholog to the human apolipoprotein L family (Fig 1A–1D). To explore the function of this transcript in developing zebrafish, we first asked whether the candidate *apol1* ortholog is expressed in a temporal manner amenable to transient assays of renal development and function. RT-PCR analysis of cDNA generated from wild-type (WT) whole-larval total RNA collected at three days post-fertilization (dpf) and 5 dpf showed expression at time points corresponding to the formation of the pronephros. Additionally, we detected *apol1* expression in flow-sorted podocyte fractions harvested from glomeruli of *pod*::NTR-mCherry adult zebrafish (Fig 1E) [28].

**Fig 2:**
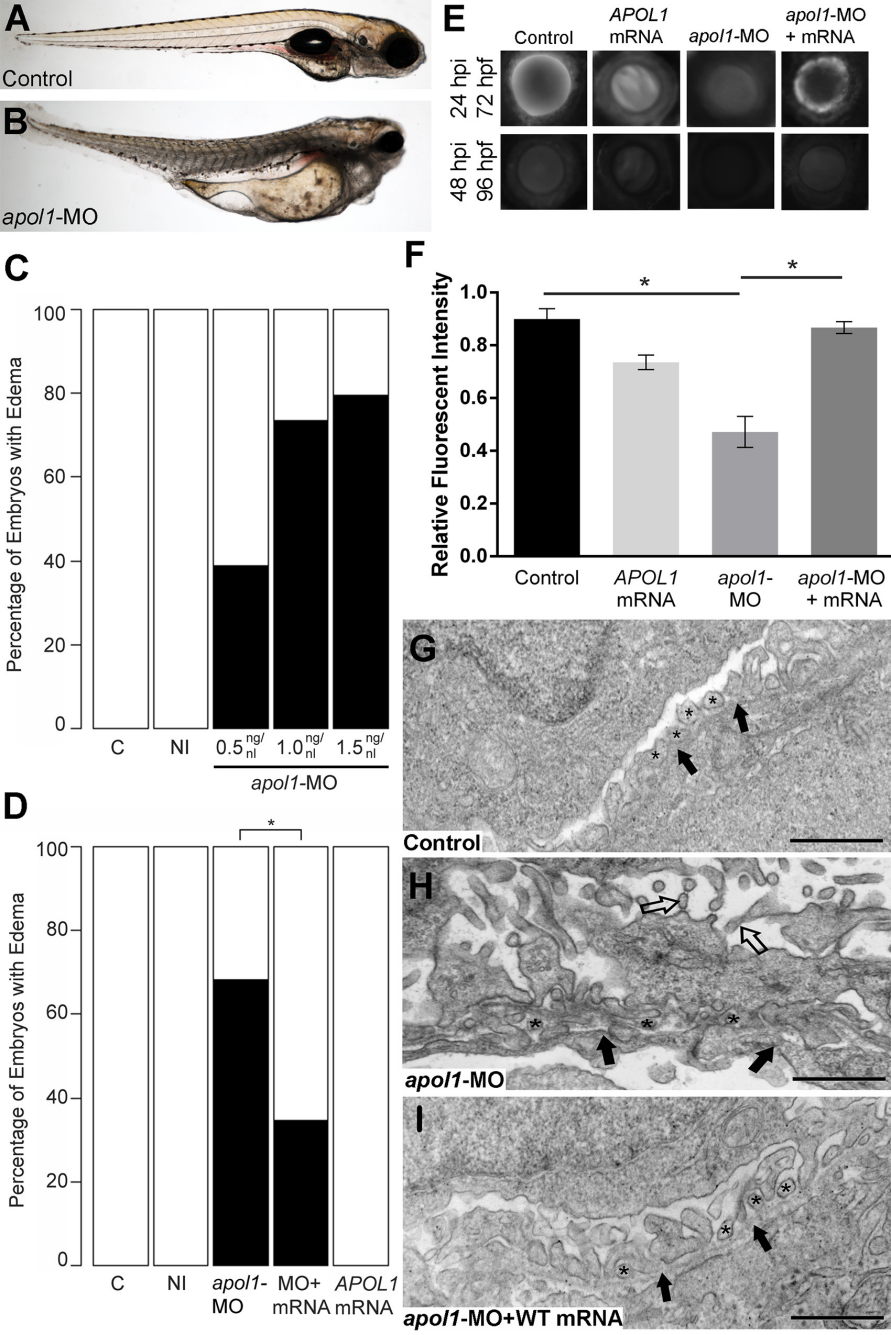
*apol1* morphant zebrafish embryos display generalized edema and glomerular filtration defects indicative of nephropathy. Representative live images of (A) sham-injected control larvae, and (B) *apol1* morpholino (MO) injected larvae at 5 dpf. *apol1* morphants display pericardial and yolk sac edema. (C) Injection of increasing doses of *apol1*-MO demonstrate dose-dependent effects when scored for generalized edema (*n* = 35–65 embryos/injection; repeated three times) compared to control larvae at 5 dpf. *apol1* morpholino injected embryos were complemented with the respective human mRNA to *APOL1* (100pg/nl) and scored for generalized edema at 5 dpf. (D) Ectopic expression of *APOL1* rescues significantly the edema phenotype observed in *apol1* morphants (1.0 ng/nl dose). We observed no significant phenotypes when *APOL1* human mRNA is injected alone. 70kDa dextran-FITC conjugate was injected into the cardiac venous sinus of 48 hpf zebrafish larvae and fluorescence intensity in the eye vasculature was measured at 24 and 48 hpi. (E) Representative eye image series of zebrafish larvae for each injection group show a relatively stable or a decrease in fluorescence intensity over time compared to sham-injected controls. (F) Bar graphs summarize the fluorescence changes observed for each injection group for *apol1* morphant larvae. Reduction in fluorescence intensity over the pupil was calculated relative to the 24 hpi time point; *apol1* morphants display increased glomerular clearance of 70kDa dextran-FITC compared to control embryos over time, indicative of compromised glomerular filtration and proteinuria. These defects were rescued significantly when MO was co-injected with orthologous human mRNA. (G-I) Compared to (G) sham-injected controls, the glomerular ultrastructure of (H) *apol1* morphant zebrafish display partial effacement of podocyte foot process (* asterisks), although the glomerular basement membrane (filled arrowheads) appears normal. Microvillus protrusions (open arrowheads) are also apparent in the urinary space. (I) Ultrastructure defects are rescued upon co-injection of human wild-type mRNA (100pg). Scale bar, 500nm. White bars, normal; black bars, edema. MO concentrations are in μg/μl, with 1nl injected into each embryo. C, sham-injected control; NI, non-injected control. Dextran values are in relative fluorescent intensity, mean ± SE. Control, sham-injected control (*n =* 29); MO, *apol1* morpholino injected (*n =* 26); *apol1-*MO+mRNA (*n =* 28). h.p.f., hours post-fertilization; h.p.i., hours post-injection. *p<0.001.

**Fig 3:**
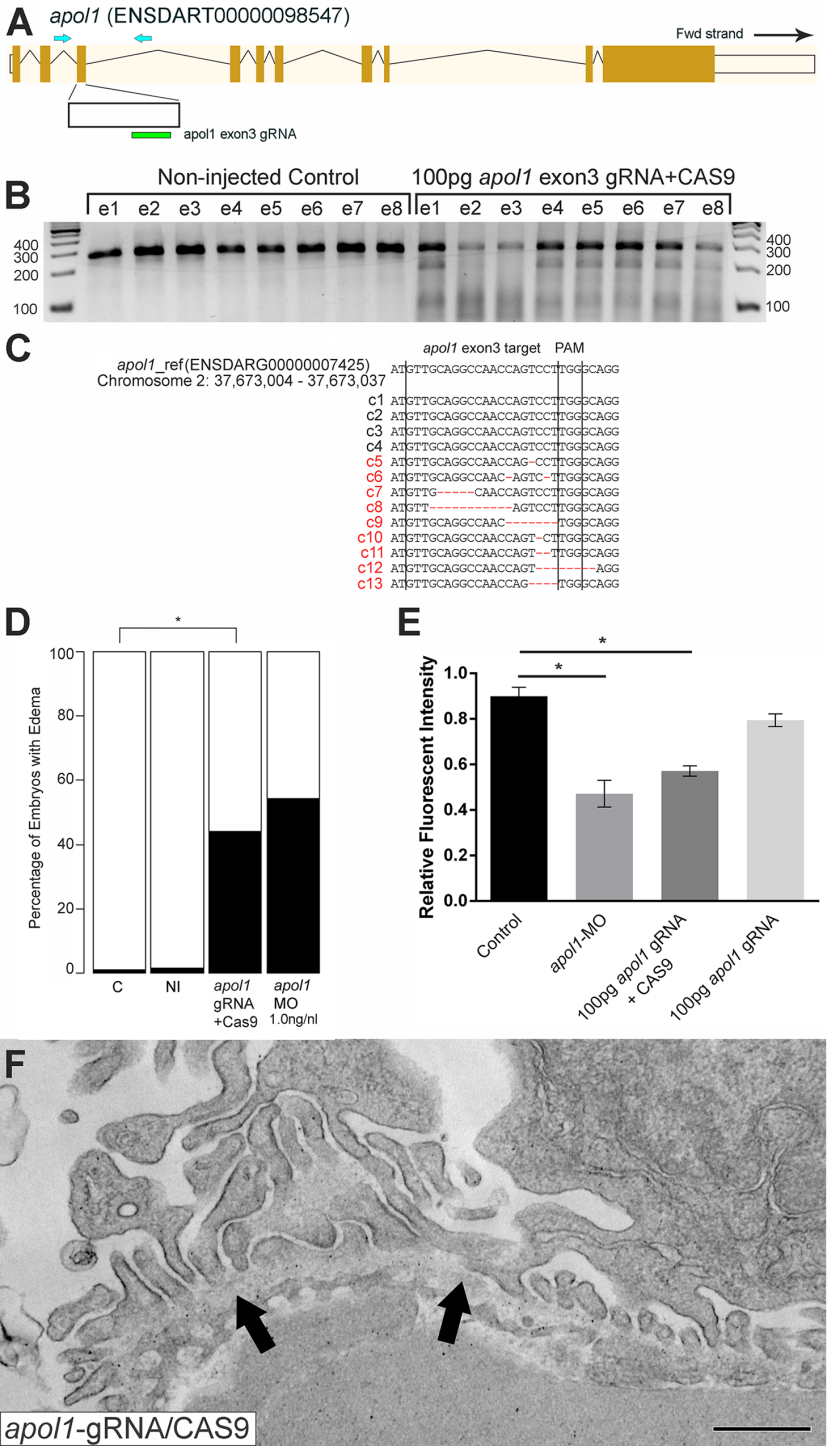
*apol1*-CRISPR F0 zebrafish embryos reproduce phenotypes observed in *apol1* morphants. (A) Schematic of the zebrafish *apol1* locus and location of the guide RNA (gRNA) target used for *apol1*-CRISPR experiments; the primers used to PCR-amplify the target region are shown (arrowheads). (B) At 1 dpf, a representative sampling of 8 founders and 8 non-injected controls were selected and subjected to T7 endonuclease 1 (T7E1) assay. The appearance of T7E1 fragments at ~180bp indicate positive gRNA targeting of exon 3 in the *apol1* locus. No T7E1 fragments were detected in non-injected control embryos. In total, 25 out of 41 founders subjected to T7E1 assay showed the presence of T7E1 fragments, indicating that ~61% of founders have insertion/deletions (indels) in the exon 3 region of *apol1*. (C) Multiple sequence alignment of *apol1* reference sequence (ENSDARG00000007425) to *apol1*-CRISPR variants generated from PCR amplification and subsequent TA cloning and sequencing of two representative *apol1*-gRNA/CAS9 injected founders. 13 PCR-cloned sequences are shown, representing four wild-type variants (c1-4) and all indel types detected among 50 PCR-clones (c5-13). Of 50 total PCR-clones sequenced, 31 showed detectable indels, representing an estimated 62% mosaicism in *apol1*-CRISPR/CAS9 injected founders. Lines mark the specific sequence targeted by the *apol1*-gRNA (exon3) and the location of the PAM recognition motif (i.e. TGG). (D) *apol1*-gRNA and CAS9 co-injected embryos were scored for edema formation at 5 dpf (n = 26–31 embryos/injection, repeated three times; *p<0.001). (E) *apol1-*gRNA and CAS9 co-injected embryos display increased glomerular clearance of 70kDa dextran-FITC compared to control embryos over time, similar to that of *apol1-*MO injected embryos (*p<0.001). Bar graphs summarize the changes for each injection group. Dextran values are in relative fluorescence intensity, mean ± SE. Control, sham-injected control (n = 19–21); *apol1*-gRNA+CAS9 (n = 11–17); *apol1*-gRNA alone (n = 13–14), repeated 2 times. (F) *apol1*-CRISPR/CAS9 injected embryos display podocyte foot process effacement at 5 dpf, similar to that of *apol1* morphant larvae. Ultrastructural defects appear less severe when compared to *apol1-*MO injected embryos, however, including less foot process effacement and the absence of microvilli in the urinary space. Filled arrowheads, glomerular basement membrane. Scale bar, 500nm.

To test the effects of *apol1* suppression, we designed a translation-blocking morpholino (MO; Gene Tools, LLC) targeting the candidate zebrafish *apol1* locus (*apol1-*MO) and we injected increasing doses into embryos at the one to four cell stage (*n* = 49–65 embryos/injection; repeated three times). Masked scoring for morphological defects at 5 dpf revealed a dose-dependent increase of the percent of larvae displaying pericardial and yolk sac edema, a phenotype that has been implicated previously in glomerular filtration defects[24, 30] (Fig 2A–2C). Co-injection of WT *APOL1* human mRNA (GenBank Accession: BC112943.1; 100 pg/nl) rescued significantly the edema caused by *apol1* suppression (p<0.0001; Fig 2D), arguing not only that the phenotype was unlikely to be a non-specific toxic effect of the MO, but also that the zebrafish locus we targeted is the ortholog of the human transcript. Importantly, co-injection of human mRNA encoding other human apolipoprotein L members (*APOL2*, *APOL3*, *APOL4*, *APOL5*, and *APOL6*) with *apol1* MO did not rescue the edema formation of *apol1* morphants (S1 Fig). Additionally, we observed a significant decrease in endogenous APOL1 protein expression in *apol1*-MO injected zebrafish embryos (p = 0.026), which is restored to normal levels upon co-injection with wild-type human *APOL1* mRNA (S2 Fig). Furthermore, as an additional test of the specificity of *apol1* perturbation to edema formation, we induced microdeletions in exon 3 of *apol1* using the CRISPR/Cas9 system[31, 32] (Fig 3A–3C). Injection of guide RNA and CAS9 protein into one-cell stage embryos reproduced the edema phenotype (scored in founders, F0) seen in *apol1* morphants (n = 26–38 embryos/injection, repeated three times; p<0.001; Fig 3D).

To test whether the generalized edema phenotype was relevant to nephropathy, we assessed the integrity of the glomerular filtration barrier in *apol1* morphants and F0 mutants as described[30]. First, we injected 70-kDa FITC-labeled dextran into the cardiac venous sinus of larvae at 48 hours post-fertilization (hpf). After injection, the eye vasculature was imaged at 24 and 48 hours post-injection (hpi; Fig 2E and 2F). We quantified the average fluorescence intensity (ImageJ) and calculated changes in intensity at 48 hpi relative to the 24 hpi measurements. *apol1* morphant larvae display a significant reduction in circulating 70-kDa dextran compared to controls (*n =* 26; p = 4.44x10^-4^; MO vs. control; Fig 2E and 2F), consistent with the occurrence of proteinuria. Importantly, this phenotype was also reproduced in *apol1* CRISPR/Cas9 larvae (Fig 3E). Upon co-injection of WT *APOL1* human mRNA, the increased dextran clearance in *apol1-*MO larvae was rescued significantly and fluorescence intensity returned to levels indistinguishable from controls (*n =* 28; p = 7.75x10^-4^, MO vs. MO + mRNA; Fig 2E and 2F).

Next, we evaluated the cellular organization and patterning of the developing glomerulus in the context of *apol1* suppression. We performed transmission electron microscopy (TEM) of ultrathin sections of zebrafish larvae at 5 dpf in WT and *apol1* morphants and mutants, with *myh9* morphants as a positive phenotypic control. In agreement with previous studies[17], *myh9* morphant larvae exhibit focal bulges and glomerular basement membrane (GBM) thickening in comparison to controls, as well as the presence of microvillus protrusions, a defining characteristic of proteinuria (S3 and S4 Figs). Notably, *apol1*-MO injected larvae display a similar glomerular ultrastructure compared with *myh9* morphants. Naked patches of GBM are apparent throughout the glomerulus, indicative of extensive podocyte effacement (Figs 2G, 2H, and S4). However, we did not observe GBM thickening as evident in *myh9*-MO injected larvae (S3 Fig). In areas in which we did observe foot process formation, podocyte protrusions were irregular and inhibited slit diaphragm development (Figs 2G, 2H, and S4). We also noted the formation of microvillus protrusion in the urinary space of *apol1* morphants. Similarly, *apol1*-CRISPR/CAS9 injected embryos display an aberrant glomerular ultrastructure, as evident by podocyte foot process effacement (Fig 3F). Co-injection of orthologous WT human mRNA in *apol1* morphants rescued these glomerular ultrastructure defects (Fig 2I). Together, these data represent compelling *in vivo* evidence implicating *APOL1* in renal function.

### Complementation of zebrafish *apol1* morphants with human *APOL1* risk alleles does not rescue kidney defects

**Fig 4:**
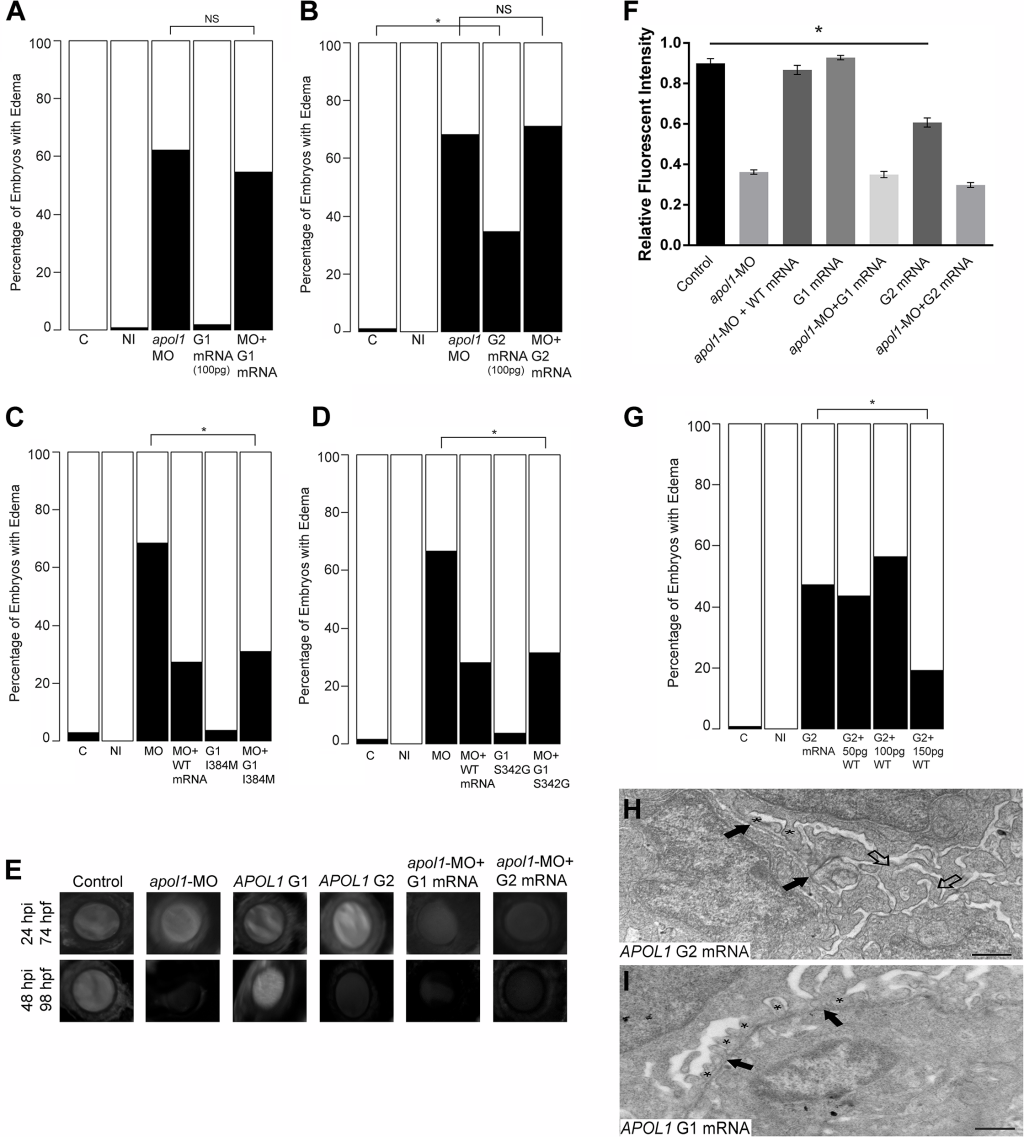
*In vivo* modeling of human *APOL1* variants associated with disease. *apol1* MO injected larvae were complemented with the respective human mRNA corresponding to *APOL1* G1 (S342G/I384M) (100pg/nl) and G2 (100pg/nl) risk variants and scored for edema formation at 5 dpf (n = 26–65 embryos/injection; repeated three times). (A, B) Neither risk variant of *APOL1* rescues significantly the edema phenotype observed in *apol1* morphants. However, when human *APOL1* G2 mRNA was injected alone (B), a significant number of embryos develop edema compared to sham-injected controls, suggesting a possible dominant-negative effect of the G2 altered protein. (C, D) *apol1* morpholino injected larvae were complemented with human mRNA corresponding to either (C) *APOL1* G1 I384M or (D) *APOL1* G1 S342G and scored for edema formation at 5 dpf (n = 48–93 embryos/injection; repeated two times). Each individual variant comprising *APOL1* G1 risk rescues significantly edema formation in *apol1* morphant embryos, suggesting that both G1 variants must be present to confer loss of APOL1 function. (E-F) *apol1* morphants co-injected with human *APOL1* G1 or G2 mRNA fail to rescue filtration defects as indicated by dextran clearance, while larvae injected with G2 mRNA alone display increased clearance over time. (G) Titration of G2 injected embryos with increasing concentrations of human WT *APOL1* mRNA show a significant reduction in edema formation of developing embryos at 5 dpf. (H) Zebrafish embryos injected with *APOL1* G2 mRNA (100pg/nl) alone display glomerular aberrations similar to that of *myh9* suppressed larvae, with microvillus protrusions present (open arrowheads), although the glomerular basement membrane appears normal (filled arrowheads). Podocyte foot processes (* asterisk) are apparent, although sparsely present. (I) Embryos injected with *APOL1* G1 mRNA (100pg/nl) alone display normal glomerular ultrastructure. Scale bar, 500nm. White bars, normal; black bars, edema. C, sham-injected control; NI, non-injected control. *p<0.05.

Initial reports associating *APOL1* variants with kidney disease in African Americans identified two independent sequence variants, termed G1 and G2, which reside in a 10-kb region in the last exon of the gene[5–7, 10]. The G1 allele consists of two nonsynonymous coding variants in perfect LD, rs73885319 and rs60910145, while the G2 variant consists of a six base pair deletion that removes amino acids N388 and Y389 (~21% and ~13% allele frequency in African Americans, G1 and G2 respectively; Fig 1D). Therefore, we evaluated the ability of each of the G1 and G2 alleles to rescue *apol1*-MO injected zebrafish larvae. *APOL1* G1 (I384M/S342G) and G2 allelic constructs were generated from a WT *APOL1* human cDNA clone, transcribed, and co-injected with *apol1*-MO in zebrafish embryos (100pg/nl). Importantly, each *APOL1* allelic construct produces a stable protein detectable by immunoblotting when co-injected with *apol1*-MO (S2 Fig). *apol1* morphants co-injected with either *APOL1* G1 (I384M/S342G) or G2 human mRNA did not display significant rescue of edema formation in developing embryos compared to *apol1*-MO injected embryos alone (Fig 4A and 4B). In addition, we also co-injected each individual G1 variant (I384M and S342G) into *apol1* morphant embryos. *APOL1* message encoding either p.I384M or p.S342G were individually able to rescue significantly the edema caused by *apol1* suppression (Fig 4C and 4D) suggesting that the *cis* effect of both variants in the same haplotype is required to confer pathogenicity. When *APOL1* G2 mRNA was injected alone, a significant number of embryos developed edema in comparison to sham-injected controls (*n =* 52–63 embryos/injection; repeated three times; p = 0.012; Fig 4B); no edema was observed with injection of 100pg *APOL1* G1 mRNA alone (Fig 4A). Additionally, dextran clearance assays demonstrated that neither *APOL1* G1 or G2 mRNA were able to rescue glomerular filtration defects caused by *apol1* suppression, while *APOL1* G2 mRNA injected alone caused significant filtration defects compared to controls (n = 12–21; p = 0.003, Control vs. G2 mRNA; Fig 4E and 4F). Finally, when we injected embryos with *APOL1* G2 titrated with increasing concentrations of *APOL1* WT mRNA, we observed a significant reduction of edema formation in developing embryos (Fig 4G) suggesting that this allele is conferring a dominant negative effect on protein function.

We also examined the glomerular ultrastructure of *apol1* morphants co-injected with either *APOL1* G1 or G2 human mRNA using TEM. However, we did not observe any noticeable improvement in glomerular ultrastructure abnormalities at 5 dpf (S5 Fig). In concurrence with our observations of gross morphological defects, embryos injected with G2 mRNA alone also display glomerular aberrations and microvillus protrusions (Fig 4H) similar to *myh9* and *apol1* morphants (Figs 2H and S4); no abnormalities were seen in larvae injected with G1 mRNA alone (Fig 4I). These data provide direct evidence for a functional consequence of the human APOL1 G1 and G2 risk alleles, and suggest that they confer loss-of-function and dominant negative effects, respectively.

### *myh9* and *apol1* interact under anemic stress to exacerbate nephropathy phenotypes

**Fig 5:**
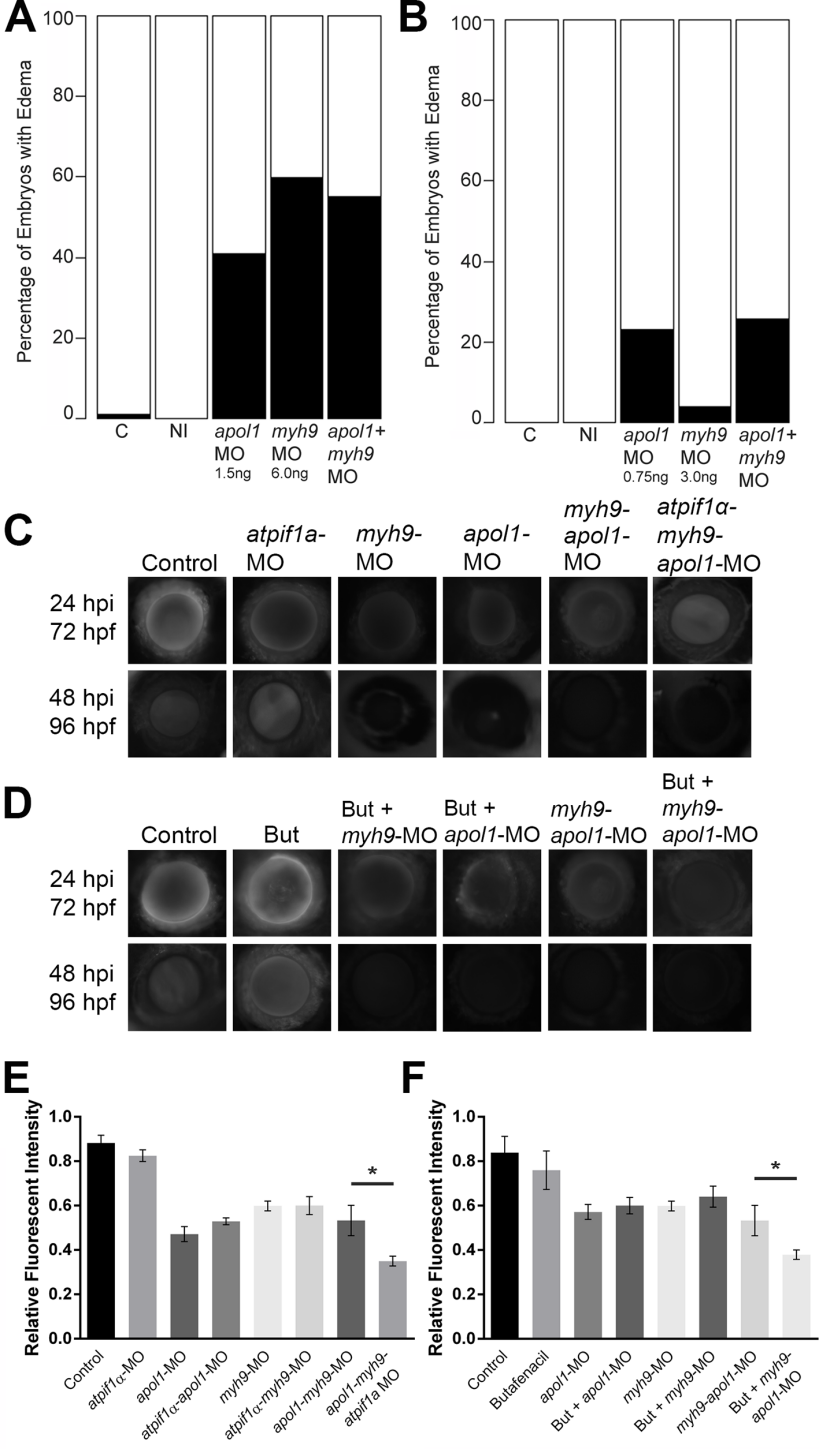
*apol1* interacts with *myh9* in an anemic context. To test for epistatic effects of *apol1* and *myh9* in zebrafish, we first co-injected both *apol1-*MO (1.0ng/nl dose) and *myh9-*MO (6.0ng/nl dose) into zebrafish larvae and scored for edema formation at 5 dpf. (n = 39–89 embryos/injection; repeated three times). However, under this co-suppression model (A, B), we observed no significantly increased edema formation compared to each MO alone. We next tested for an interaction between *apol1* and *myh9* in the context of *atpif1a* suppression, predicting that the added stress of anemia would mimic our initial observations in sickle cell disease patients. 70kDa dextran-FITC conjugate was injected into the cardiac venous sinus of 48 hpf zebrafish larvae and fluorescence intensity in the eye vasculature was measured at 24 and 48 hours later. (C) Representative eye image series of zebrafish embryos for each injection group show relatively stable or decreased fluorescence intensity over time. (E) Bar graphs summarize the changes observed for each injection group. Zebrafish embryos injected with all three MOs show a significant increase in dextran clearance from the vasculature compared to co-suppression of *apol1* and *myh9*. (D, F) These data are reproduced using butafenacil induced anemia (0.195 μM in embryo media, treated at 48 hpf). Dextran values are in relative fluorescence intensity, mean ± SE. Control, sham-injected control (*n =* 19); *atpif1a* MO injected (*n =* 14); *apol1-*MO+*myh9*-MO (*n =* 12); *apol1-*MO+*myh9*-MO+*atpif1a*-MO (n = 11); Butafenacil (n = 48); But+*myh9-*MO+*apol1-*MO (n = 18). hpf, hours post-fertilization; hpi, hours post-injection. *p<0.001.

Although recent studies have provided statistical evidence implicating *APOL1* variation in nondiabetic nephropathies[7, 33, 34], *MYH9* risk variants are still associated with chronic kidney disease (CKD) in non-African American populations[35] and in sickle cell disease nephropathy[5]. As such, our group and others have hypothesized that these genes may be co-regulated to induce nephropathy risk; in fact, when we modeled glomerular filtration rate in sickle cell patients as a function of the previously reported *MYH9* risk haplotype and an *APOL1* recessive model, we observed a significant interaction between the two genes[5]. Therefore, we tested for functional interaction effects between *apol1* and *myh9* in zebrafish, an experimentally tractable model for investigating additive and synergistic effects[36–40]. First, we co-injected both *apol1*-MO and *myh9-*MO into embryos and we scored for gross morphological defects at 5 dpf. Under this co-suppression model, we observed no significant differences in edema formation when compared to batches injected with either MO alone (Fig 5A), even when individual MO concentrations were reduced to subeffective doses (Fig 5B). Next, we tested the possibility that suppression of either *apol1* or *myh9* in zebrafish could be rescued significantly by the co-injection of the reciprocal human mRNA. *myh9*-MO was co-injected with human *APOL1* WT mRNA (100pg/nl) and *apol1*-MO was co-injected with human *MYH9* WT mRNA (100pg/nl). However, we were unable to rescue the suppression phenotypes of either *apol1* or *myh9* with the human mRNA of the reciprocal gene (S6 Fig).

Our hypothesis for an interaction between *APOL1* and *MYH9* was based on data derived from SCD patients. Thus, we posited that *myh9* and *apol1* may only interact under additional biologic stress, such as anemia or hemolysis. Accumulating evidence suggests that both anemia and hemolysis, which are key features of SCD pathophysiology, impact renal function; in particular, hemolysis appears to be associated with both microalbuminuria and hyperfiltration[41, 42]. While a zebrafish model of SCD does not exist currently, suppression of ATPase inhibitory factor 1 (*atpif1α*), a mitochondrial protein, produces profound anemia in zebrafish by interfering with heme synthesis through decreased catalytic efficiency of ferrochelatase[43]. The resultant effect of low hemoglobin and hematocrit stresses the kidney because of the organ’s high oxygen consumption. Consistent with the original report[43], we observed a dose-dependent reduction in hemoglobin with increasing concentrations of the *atpif1a* MO (*atpif1α-*MO), as measured by o-dianisidine staining of whole MO-injected larvae at 4 dpf. Strikingly, we found a significantly more severe nephropathy phenotype in an anemic context as indicated by accelerated dextran clearance, with co-suppression of *apol1* and *myh9* under *atpif1α*-MO induced anemia (n = 12–19 embryos/injection; p<0.001 for *myh9/apol1* MOs vs. *myh9/apol1/atpif1a* MOs; Fig 5C and 5E). Importantly, neither morphant alone resulted in a more severe phenotype under *atpif1α*-MO induced anemia (e.g. *myh9*-MO vs. *myh9-atpif1α-*MO; p = 0.78; or *apol1-*MO vs. *apol1-atpif1α*-MO; p = 0.90; Fig 5E). Furthermore, these observations were reproducible using an independent and non-genetic induction of anemia. Butafenacil, an inhibitor of protoporphyrinogen oxidase, causes loss of hemoglobin following exposure during early zebrafish development[44]. In a butafenacil-induced anemic context (0.195 μM treatment at 48 hpf), we observed a similar effect upon co-suppression of *apol1* and *myh9* (n = 17–23 embryos/injection; p<0.001 for *myh9/apol1* MOs vs. *myh9/apol1* + 0.195 μM butafenacil; Fig 5D and 5F).

### *APOL1* G2 (*del*:*N388Y389*) modulates *myh9* expression *in vivo*

**Fig 6:**
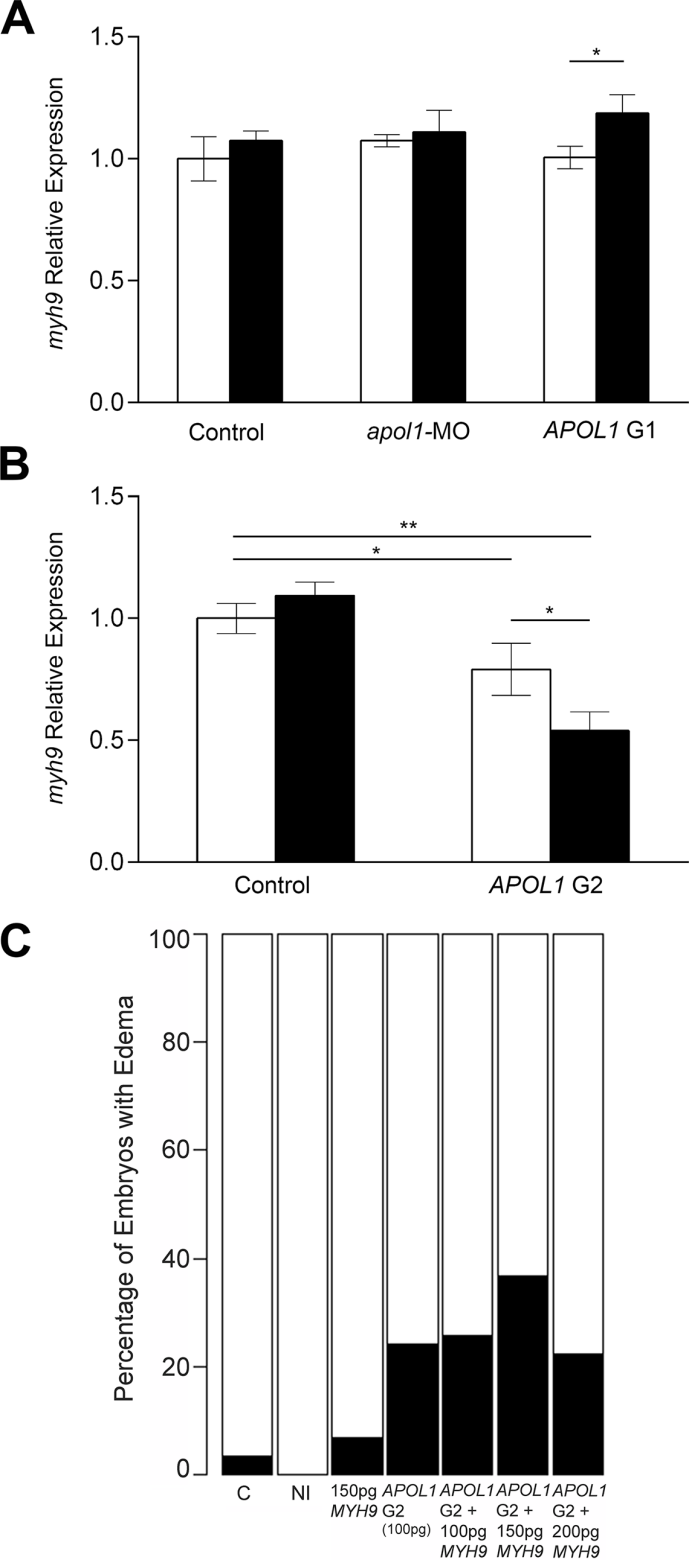
*myh9* expression in the context of *apol1*/*APOL1* modulation. Zebrafish embryos were injected with either *apol1*-MO (1.0ng/nl dose), *APOL1* G1 (S342G:I384M) mRNA (100pg), or *APOL1* G2 (100pg) mRNA alone, in the absence (white bars) or presence (black bars) of *atpif1*α-MO. Total RNA at 5 dpf or 3 dpf (*APOL1* G2/*atpif1α*-MO embryos did not survive to 5 dpf) was extracted and reverse-transcribed with random primers to obtain whole-embryo cDNA. *myh9* expression was determined by quantitative real-time PCR and relative expression was calculated against *actb1*. (A) *apol1*-MO injected embryos do not display any significant changes in *myh9* expression compared to sham-injected control embryos. Additionally, *APOL1* G1 expression does not alter *myh9* expression alone, however, under *atpif1*α-induced anemia, we observe an increase in *myh9* expression. (B) *APOL1* G2 expression results in a significant decrease in *myh9* expression compared to sham-injected control embryos, suggesting that the altered APOL1 protein may regulate *myh9 in vivo*. (C) Co-injection of *APOL1* G2 (100pg) and human WT *MYH9* (n = 31–60; repeated two times), does not rescue edema formation caused by *APOL1* G2 expression in 5 dpf larvae, suggesting that the interaction between *APOL1* G2 and *MYH9* may be indirect. Relative expression values are mean ± SE in triplicate with two biological replicates. * = p<0.05; ** = p<0.01.

To dissect further the possible genetic interactions between *myh9* and *apol1*, we tested whether suppression of endogenous *apol1* or ectopic expression of mutant human *APOL1* could alter expression of *myh9* in zebrafish embryos. We monitored *myh9* expression in zebrafish larvae using quantitative real-time PCR in the context of *apol1* suppression, and G1 or G2 expression, as well as *apol1*/*APOL1* modulation in conditions of anemia induced by *atpif1α*-MO injection at 5 dpf (Fig 6A) and 3 dpf (Fig 6B). We observed a significant decrease in *myh9* expression when zebrafish embryos were injected with the proposed dominant-negative *APOL1* G2 allele alone (21% reduction; p = 0.043; Fig 6B), suggesting that the mutant protein may be suppressing *myh9*, either directly or indirectly, to induce nephropathy. Furthermore, zebrafish embryos co-injected with *APOL1* G2 mRNA and *atpif1α*-MO display an even greater reduction in *myh9* expression compared to controls (46% reduction; p = 0.0013; Fig 6B), and a significant reduction of *myh9* expression compared to *APOL1* G2 mRNA alone (p = 0.0297; Fig 6B), suggesting that the altered APOL1 (p.Asn388_Tyr389del) protein has a more pronounced effect on *myh9* expression in the context of anemic stress. We also observed a significant increase in *myh9* expression in *APOL1* G1/*atpif1α*-MO vs. *APOL1* G1 injected embryos (Fig 6A), however, neither of these conditions induced nephropathy. To determine whether this effect was specific to *myh9* or was a general effect on transcripts expressed in the glomerulus, we also assessed expression levels of other nephropathy-associated genes during *apol1/APOL1* modulation and *atpif1α* induced anemia. We observed no significant differences in expression of genes implicated in familial focal segmented glomerulosclerosis, including *anln*[45], *trpc6b*[46], and *wt1a*[47] upon *apol1*/*APOL1* modulation (S7 Fig), suggesting that *APOL1* G2 regulation may be specific to *myh9*.

Based on the observations that *APOL1* G2 expression has the ability to decrease *myh9* expression *in vivo*, we next attempted to rescue *APOL1* G2 defects by co-injecting human WT *MYH9* mRNA. We injected a constant amount of *APOL1* G2-encoding message (100pg) with increasing amounts of human *MYH9* mRNA (100pg, 150pg, and 200pg) and scored larvae live for generalized edema at 5dpf. However, we did not observe a significant reduction of edema in *APOL1* G2/*MYH9* co-injected embryos (Fig 5C), suggesting that compensation with *MYH9* message alone is not sufficient to account for the deleterious effects of the G2 variant, possibly because APOL1 G2 has a *trans* effect on other loci in the genome or is acting to perturb cellular pathways[20].

## Discussion

In recent years, multiple lines of statistical evidence have implicated the *MYH9*/*APOL1* locus on chromosome 22q12.3 with nondiabetic end-stage renal disease, focal segmental glomerulosclerosis, HIV-associated nephropathy, lupus nephritis, SCDN, and diabetic nephropathy in patients of recent African ancestry and European Americans[5–10, 33, 35, 48–50]. Additionally, *APOL1* has been associated with an increased burden of cardiovascular disease in African Americans participating in the Jackson Heart Study[51]. Compelling statistical evidence in human cohorts points to the G1 and G2 alleles of *APOL1*, rather than *MYH9* variation, as the most likely contributors to nephropathy risk. Nonetheless, functional studies of the *MYH9* locus provide biological evidence for its role in the kidney, including perturbed glomerular development in *myh9* morphant zebrafish[14–17]. Here, we have identified a functional ortholog of human *APOL1* in zebrafish and, using transient genetic manipulation, provide functional evidence demonstrating *apol1* involvement in both kidney development and filtration.

Although the human APOL gene cluster has undergone recent natural selection in primates[26, 27], we report the identification of a functional *APOL1* ortholog in the zebrafish genome and its implication in renal function. Specific detection of the zebrafish *apol1* protein product with the human APOL1 antibody, rescue of kidney defects in *apol1* morphant embryos with human *APOL1* mRNA, as well as recapitulation of renal phenotypes with an *apol1*-CRISPR/CAS9 F0 mutant, provide evidence that zebrafish *apol1* is indeed functionally relevant to its human ortholog with respect to its role in the glomerulus. Furthermore, no other human mRNA in the human apolipoprotein L family ameliorated kidney defects induced by *apol1* knockdown, supporting further its functional orthology to human *APOL1*. Nonetheless, it is unclear whether the zebrafish APOL1 protein serves all functions of its human counterpart, especially given the lack of a secretory domain in the zebrafish APOL1 peptide (Fig 1A).

Suppression and genome-editing of *apol1* in zebrafish and three independent phenotypic scoring paradigms support a role for *apol1* in nephropathy; we observed severe edema formation with concomitant glomerular filtration defects and severe podocyte loss. Complementation of *apol1* suppression with *APOL1* CKD risk alleles (G1 and G2) failed to ameliorate these observed defects. Notably, complementation of each individual variant of the G1 haplotype (I384M and S342G) rescued significantly nephropathy phenotypes caused by *apol1* suppression, suggesting that both variants must be present in *cis* to confer risk. This is concordant with initial reports on the lytic potential of APOL1 recombinant proteins on *T*. *b*. *rhodesiense*, in which APOL1 variants with either S342G or I384M alone were less lytic than if both were present together[6].

Strikingly, injection of human *APOL1* G2 mRNA alone resulted in significant edema formation in 5dpf zebrafish larvae as well as perturbed glomerular filtration and ultrastructural defects. Our expression data suggest that this could arise from *myh9* suppression induced by the altered APOL1 protein harboring the G2 variant. The G2 deletion lies in the SRA-binding domain of APOL1 (Fig 1B and 1D). Therefore it is plausible that disruption of this region of the protein may either prohibit proper binding of APOL1 to its usual partners, or perhaps permit new interactions that induce nephropathy. Further studies are needed to elucidate the functional impacts of the altered APOL1 protein to nephropathy. We also report for the first time functional evidence of a genetic interaction between *myh9* and *apol1*. Intriguingly, this interaction was only observed in the presence of anemic stress, consistent with our previous genetic association findings in human SCD patients[5].

An immediate question remains regarding the mechanism by which *apol1* suppression is inducing kidney injury. Early studies revealed *APOL1* mRNA expression in the placenta, lung, and liver, with specific cell-type expression found in endothelial cells and possibly macrophages[26]. More recent studies, however, have characterized the cellular localization of APOL1 in human kidney sections to podocytes, proximal tubules, and arteriolar endothelial cells[18]. These data are consistent with our observation of *apol1* morphants and mutants exhibiting extensive podocyte loss and suggest that *apol1* is necessary for the development and/or maintenance of glomerular podocytes. Interestingly, it has been shown that APOL1 may cause toxic renal effects through programmed cell death pathways leading to glomerulosclerosis[52, 53]. Thus, *apol1* suppression could dysregulate autophagic pathways, causing podocyte malformation, thereby promoting the susceptibility of the pronephros to glomerular injury.

Initial studies implicating *MYH9* in nondiabetic nephropathy failed to identify coding variants associated with renal outcome[8, 9], and since the nearby nonsynonymous variants identified in *APOL1* provided stronger statistical association[5–7], it was hypothesized that *APOL1* variation represents the true attribution to renal disease risk. In fact, it has been shown in multiple studies that controlling for the *APOL1* risk alleles (G1-G2) attenuates significantly the effect of *MYH9* SNPs[6, 33]. However, recent reports still demonstrate statistical association of *MYH9* in nondiabetic nephropathy[5, 35] and previous *in vivo* modeling studies provide further evidence for the role of *Myh9* in glomerular development and glomerulosclerosis[14–17]. As such, our group and others have postulated that complex genetic models may exist in this region, including the possibility of *MYH9*-*APOL1* gene interaction[5, 10]. Our observation of exacerbated glomerular filtration in the context of anemic stress provides biological evidence in support of this hypothesis. Because knockdown of each of *myh9* and *apol1* independently impairs proper pronephric development and filtration, it is plausible that their encoded proteins are functioning in separate pathways to induce kidney dysfunction. However, these effects only appear to become additive under an additional stress (anemia). The associated variants alone may not be sufficient to induce nephropathy progression, while under low hemoglobin and hematocrit levels, additive effects between *MYH9* and *APOL1* may become apparent and result in a more drastic reduction in renal function, along with the observed significantly high early mortality rates among SCD nephropathy patients[21, 22, 41, 54].

Furthermore, we provide evidence suggesting that the functional consequences of *APOL1* variation may not be acting in a strictly recessive manner as had been previously suggested[5–7, 55]. Our data demonstrate that *APOL1* G1 (I384M/S342G) confers loss of proper APOL1 function in the developing zebrafish kidney, while *APOL1* G2 is acting in a dominant-negative manner to induce nephropathy, possibly through suppression of *myh9*. These data indicate that the risk conferred by the *APOL1*/*MYH9* locus is likely to be governed by a more complex model than recessive patterning as suggested previously.

In summary, our study demonstrates the essential role of both *apol1* and *myh9* in the development of the pronephric glomerulus and proper renal filtration in zebrafish. We report comprehensive *in vivo* causal evidence of *apol1* involvement in kidney decline, and we provide the first *in vivo* evidence of a potential dominant-negative effect of the *APOL1* G2 allele. Further, we have shown that the presence of the G2 allele decreases significantly the expression of *myh9*. Similar to the common haplotype on 10q26 that influences age-related macular degeneration underscored by complex regulatory events of neighboring genes *ARMS2* and *HTRA1*, our data highlight further the importance of comprehensive evaluation of functional consequences at a susceptibility locus[56]. Taken together, these data provide essential biological insight into the mechanisms by which *MYH9* and *APOL1* confer disease risk and progression in human nondiabetic nephropathies.

## Materials and Methods

### Zebrafish stocks

We maintained WT zebrafish stocks (Ekkwill, Ekkwill x AB F1 outcross, or *pod*::NTR-mCherry[28] according to standard zebrafish husbandry procedures. Embryos were obtained from natural matings of adult fish.

### Morpholino oligonucleotide-mediated knockdown and human mRNA complementation

Complementation assays were designed essentially as described[57]. Briefly, a MO was designed by Gene Tools, LLC (Philomath, OR) to target the translation initiation site of zebrafish *apol1* (NM_001030138) (*apol1*-MO), (5’-AGTCGTCCAGCCATTCCATGAGGGT-3’). A translation-blocking morpholino (MO) targeting zebrafish *myh9* and a splice-blocking MO targeting zebrafish *atpif1a* were described previously[17, 43]. *APOL1* G1 and G2 allelic constructs were synthesized from a WT *APOL1* human ORF clone (GenBank: BC112943) using site-directed mutagenesis (Stratagene, QuikChange II), subsequently transcribed (mMESSAGE mMACHINE, Life Technologies, Ambion) into capped mRNA and co-injected with *apol1*-MO into zebrafish embryos at the one-to-four cell stage (WT, 100pg/ nl; G1, 100pg/nl; G2, 100pg/nl). Controls were injected with phenol red. A WPI pneumatic pico pump microinjector was used for MO and mRNA injection to deliver 1 nl/embryo. After injection, embryos were maintained at 28°C in embryo medium.

### Dextran microinjection and time-lapse filtration scoring

48 h.p.f. larvae were anesthetized in 1.0% tricaine and placed laterally in agarose wells. 70 kDa FITC-conjugated dextran (LifeTechnologies, 3.0nl/embryo) was injected into the cardiac venous sinus and larvae were transferred to embryo medium for recovery after injection. The eye vasculature of individual fish was imaged at 24, and 48 hours after dextran injection using a Nikon AZ100 fluorescent microscope and Nikon NIS Elements AR software. The average fluorescence intensity was measured across the eye (ImageJ) and changes in intensity relative to the 24 h.p.i measurements were calculated for comparison. GraphPad Prism version 6.03 (GraphPad Software, San Diego, CA) was used for statistical analysis of relative intensity.

### Fluorescence-activated cell sorting (FACS)

Glomeruli from *pod*::NTR-mCherry adult zebrafish were manually dissected and dissociated in 0.5% trypsin/collagenase. Dissociated cells were then filtered through a 70μm strainer and filtered again through a 30μm strainer. Cell-sorting was done on a Beckman Coulter Astrios instrument for mCherry (610nm). Sorted cells were placed in RLT Buffer (Qiagen) and RNA was extracted using the RNeasy Micro Kit (Qiagen).

### Reverse transcription and quantitative real-time PCR (qRT-PCR)

Total RNA from zebrafish embryos was extracted with TRIzol Reagent (Life Technologies) and cDNA was reverse transcribed using QuantiTect Reverse Transcription Kit (Qiagen). The following primers were used for amplification: *actb1*, Fwd: TTGTTGGACGACCCAGACAT, Rev: TGAGGGTCAGGATACCTCTCTT; *nphs2*, Fwd: CCTTCGCTAGCATTCCAGAC, Rev: GCAGCTCTGGAGGAAGATTG; *wdr81*, Fwd: ATGGAGAGAAAAACATGGAGGA, Rev: AAGGAGAAAACCTGGAAGAACC; *apol1*, Fwd: GACTTTCGATTAAGTGAAACTCAGAGAGA, Rev: GTTATGGTAGCTACACCTCCCACAGCGCTG; *myh9* (qRT), Fwd: GGAAAAACCGAAAACACCAA, Rev: CAATATTGGCTCCAACGATGT; *anln* (qRT), Fwd: TTTGACCTTCACCACCACATT, Rev: TTTGGTGTGATTGCCTTTGA; *wt1a* (qRT), Fwd: ATGGCCAAACTGTCAGAAGAA, Rev: TTATTTCCTGCCGTTTCTGTG; *trpc6b* (qRT), Fwd: GGCACCATGAGCCAGAGCCCGGCGTTCGGG, Rev: CTAAGGTGGGCCCATTGGCACTTAAGAAAA. qRT-PCR was performed on a ABI Prism 7900HT instrument and cycle threshold values were computed using SDS 2.3 software (Applied Biosystems). Relative expression was calculated against *actb1* in each sample and compared against sham-injected controls to determine significant differences in expression.

### Transmission electron microscopy of glomerular ultrastructure

5 dpf embryos were anesthetized in 1.0% tricaine and then fixed in 4.0% gluteraldehyde in 0.1M Na_2_PO_4_ buffer containing 0.12mM CaCl_2_ at 4°C overnight. Fixed larvae were washed in 1X PBS, washed in 1X phosphate buffer, postfixed in 2% osmium tetroxide for 2 hours, and dehydrated through a graded acetone series. Embedding was performed with Epoxy 812. Sections were cut on a Leica-Reichert Ultracut E ultramicrotome and semithin sections (1.0μm) were collected and stained with toluidine blue. 90nm ultrathin sections were placed on copper grids and contrasted with 4.0% uranyl acetate for 10 minutes. Grids were incubated in lead citrate (Reynolds Lead) for 3 minutes and then examined on a Phillips CM12 electron microscope. Images were taken with an AMT XR61 camera.

### Genome-editing of the *apol1* locus using the CRISPR/CAS9 system

*apol1* gRNA was produced by synthesizing and annealing two oligonucleotides, gRNA F: TAGGGTTGCAGGCCAACCAGTCCT and gRNA R: AAACAGGACTGGTTGGCCTGCAAC. The annealed oligos were then ligated to a T7cas9sgRNA2 vector by performing the ligation and digestion in a single step in a thermal cycler as described [31]. 2 μL of the reaction was used for transformation. Prior to transcription, the gRNA vector was linearized with *Bam*HI. gRNA was transcribed using the MEGAshortscript T7 kit (Life Technologies, AM1354) and purified using alcohol precipitation. A total of 100pg of *apol1* gRNA and 200pg of CAS9 protein (PNA Bio) was co-injected into individual cells of one-cell stage embryos. For T7 endonuclease I assay, genomic DNA was prepared from 1 dpf embryos as described [58]. A short stretch of the genomic region (~270–280 bp) flanking the *apol1* gRNA target site was PCR amplified from the genomic DNA (Fwd: TGTGTGAAGGATGCATTTGTT, Rev: TGGGATAATGTATGGGAGAATG). The PCR amplicon was then denatured slowly and reannealed to facilitate heteroduplex formation. The reannealed amplicon was then digested with 5 units of T7 endonuclease I (New England Biolabs) at 37°C for 45 minutes. The samples were resolved by electrophoresis through a 3.0% agarose gel and visualized by ethidium bromide staining.

### Western blot

Whole embryo protein lysates were collected at 2 dpf by homogenizing anesthetized embryos immersed in RIPA Buffer (50 mM Tris, 150 mM NaCl, 0.1% SDS, 0.5% sodium deoxycholate, 1% Triton X 100, protease inhibitor (Roche, cat. no. 11697498001)). 100 mg protein was loaded into individual wells of a Mini-PROTEAN TGX Precast Gel (Bio-Rad) and a western blot was performed as described [59]. Blots were incubated overnight at 4°C with anti-APOL1 antibody (1:1000; Abcam, EPR2907, ab108315). The membranes were subsequently washed in PBST (0.1% Tween 20) and incubated for 1 hour at room temperature with anti-rabbit IgG conjugated to horseradish peroxidase (1:20,000; GE Healthcare, NA934V). ACTIN antibody (1:1000, Santa-Cruz, cat. no. sc-8432) was used as a loading control.

### Ethics statement

All animal protocols were reviewed and approved by the Duke University Institutional Animal Care & Use Committee (IACUC; protocol A229-12-08).

## Supporting Information

S1 Fig Complementation of zebrafish *apol1* morphants with other members of the human *APOL* gene cluster. (A-E) Human mRNA corresponding to *APOL2*, *APOL3*, *APOL4*, *APOL5*, and *APOL6* (100pg/nl) were each co-injected with *apol1* MO and scored for edema at 5 dpf. Ectopic expression of each of the other members of the human *APOL* gene cluster was unable to rescue significantly the edema formation in developing embryos co-injected with *apol1* MO. (F) We observed a novel body axis phenotype in embryos injected with either *APOL3* or *APOL5* alone, although this did not seem to be relevant to kidney dysfunction. White bars, normal; black bars, edema; grey bars, adverse. C, sham-injected control; NI, non-injected control; n = 32–68 embryos/injection batch; masked scoring.
(TIF)

S2 Fig Characterization of APOL1 protein levels in *apol1*-MO and *APOL1* RNA-injected embryos. Protein lysates from zebrafish embryos injected with *apol1*-MO (1.0ng/nl) alone or co-injected with either wild-type, G1, or G2 *APOL1* human mRNA (100pg) were isolated from 2 dpf embryos. (A) APOL1 protein levels were assessed by Western blot (Abcam EPR2907) and (B) pixel intensity normalized to ACTIN was calculated for comparison. (A-B) Embryos injected with translation-blocking *apol1*-MO display a significant reduction in APOL1 protein expression compared to non-injected controls, suggesting cross-reactivity with zebrafish APOL1 and efficiency of the *apol1* MO to block translation. Protein levels are restored to control levels upon co-injection of wild-type, G1, or G2 *APOL1* human mRNA. Blot shown is a representation of four independent experiments. Lane 1, non-injected control; Lane 2, *apol1*-MO injected; Lane 3, *apol1*-MO + wild-type *APOL1* human mRNA; Lane 4, *apol1*-MO + G1 *APOL1* human mRNA; Lane 5, *apol1*-MO + G2 *APOL1* human mRNA. *p = 0.026.
(PNG)

S3 Fig *myh9* suppression and complementation in developing zebrafish embryos. We recapitulated data reported by Müller *et al*. for experimental comparison [17]. (A-B) Representative live images of sham-injected control and *myh9* morpholino (MO) injected larvae at 5 dpf. (C) Injection of increasing doses of *myh9* MO demonstrate dose-dependent effects when scored for generalized edema compared to control embryos at 5 dpf. (E-F) *myh9* morphants also display filtration defects indicated by significantly increased dextran clearance. (D-F) Co-injection of wild-type human *MYH9* mRNA (100pg/nl) significantly rescues edema formation and filtration defects observed in *myh9* morphants. (G) As reported previously by Müller *et al*., *myh9* morphants display ultrastructure abnormalities, including glomerular basement membrane thickening and the presence of microvillus protrusions in the urinary space. (H) These ultrastructural defects are rescued upon co-injection of wild-type human *MYH9* mRNA (100pg). White bars, normal; black bars, edema; n = 49–70 and n = 13–29 embryos/injection batch for gross morphological scoring and glomerular filtration assays, respectively; *p<0.05; **p<0.01; ***p<0.001; filled arrowheads, glomerular basement membrane; open arrowheads, microvillus protrusions.
(TIF)

S4 Fig Further characterization of *apol1* and *myh9* morphant glomerular ultrastructure. Transmission electron microscopy of zebrafish larval glomeruli injected with either (A) *apol1*-MO or (B) *myh9*-MO were imaged at 5 dpf using a low magnification (direct mag = 4400X) to characterize long stretches of the glomerular basement membrane (GBM). Comparatively, *apol1* and *myh9* morphants display similar abnormalities, including podocyte disorganization and effacement, as well as the presence of microvillus protrusions. However, *myh9* morphants display a thickened GBM that is not apparent in *apol1*-MO injected larvae, while *apol1* morphants appear to have a higher degree of podocyte effacement compared to *myh9* morphants. (C) Zebrafish larvae injected with *apol1* CRISPR/CAS9 display a similar glomerular ultrastructure compared to *apol1* morphants at 5 dpf. Filled arrowheads, glomerular basement membrane. Scale bar = 500nm.
(TIF)

S5 Fig Glomerular ultrastructure of *apol1* morphants complemented with human risk alleles. Transmission electron microscopy of zebrafish larval glomeruli imaged at 5 dpf. (A, B) *apol1* morphants complemented with risk alleles, G1 and G2 do not rescue the observed defects caused by *apol1* suppression, with naked patches of glomerular basement membrane and microvillus processes apparent. *, microvillus protrusions; filled arrowheads, glomerular basement membrane. Scale bars, 500nm.
(TIF)

S6 Fig Complementation of *apol1* and *myh9* morphants with each respective reciprocal human wild-type mRNA. (A) *apol1*-MO was co-injected with human WT *MYH9* mRNA (100pg/nl) and (B) *myh9*-MO was co-injected with human WT *APOL1* mRNA; embryos were scored for edema formation at 5 dpf (n = 25–66 embryos/injection for *apol1*-MO/*MYH9* RNA and n = 32–46 embryos/injection for *myh9*-MO/*APOL1* RNA); each repeated three times.
(TIF)

S7 Fig *apol1*/*APOL1* modulation effect on causal familial Focal Segmental Glomerulosclerosis (FSGS) genes. Zebrafish embryos were injected with either *apol1*-MO (1.0ng/nl dose), *APOL1* G1 (S342G:I384M) mRNA (100pg), or *APOL1* G2 (100pg) mRNA alone, in the absence (white bars) or presence (black bars) of *atpif1*α-MO. Total RNA at 5 dpf or 3 dpf (*APOL1* G2/*atpif1α*-MO embryos did not survive to 5 dpf) was extracted and reverse-transcribed with random primers to obtain whole-embryo cDNA. (A-B) *anln*, (C-D), *wt1a*, (E-F) or *trpc6b* expression was determined by quantitative real-time PCR and relative expression was calculated against *actb1*. We observed no significant differences in expression in any of the FSGS-associated genes tested under *apol1*/*APOL1* modulation, suggesting that *APOL1* G2 regulation may be specific to *myh9*. White bars = normal; black bars = *atpif1α-*induced anemia. Relative expression values are mean ± SE in triplicate with two biological replicates.
(TIF)
