# Correction: *In vivo* Modeling Implicates *APOL1* in Nephropathy: Evidence for Dominant Negative Effects and Epistasis under Anemic Stress

**DOI:** 10.1371/journal.pgen.1005459

**Published:** 2015-09-17

**Authors:** Blair R. Anderson, David N. Howell, Karen Soldano, Melanie E. Garrett, Nicholas Katsanis, Marilyn J. Telen, Erica E. Davis, Allison E. Ashley-Koch

The following information is missing from the Funding section: This study was supported by National Institutes of Health, P50 DK096415: to NK.
