# Supplementary figures and images for: In vivo Modeling Implicates APOL1 in Nephropathy: Evidence for Dominant Negative Effects and Epistasis under Anemic Stress
Source: PLoS Genet. 2015 Jul 6;11(7):e1005349. doi: 10.1371/journal.pgen.1005349 (PMC4492502; doi:10.1371/journal.pgen.1005349)

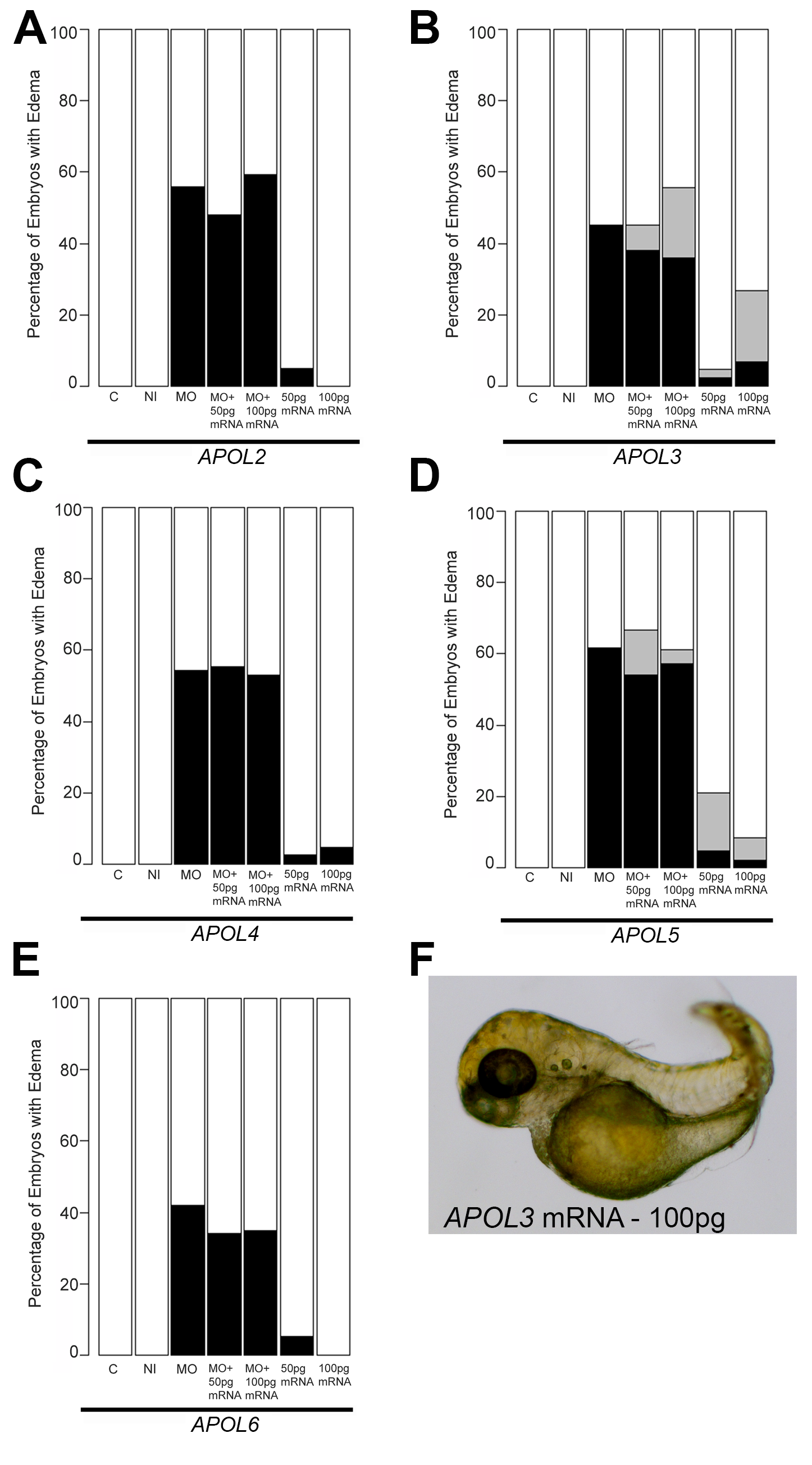

Supplement: S1 Fig — (A-E) Human mRNA corresponding to APOL2, APOL3, APOL4, APOL5, and APOL6 (100pg/nl) were each co-injected with apol1 MO and scored for edema at 5 dpf. Ectopic expression of each of the other members of the human APOL gene cluster was unable to rescue significantly the edema formation in developing embryos co-injected with apol1 MO. (F) We observed a novel body axis phenotype in embryos injected with either APOL3 or APOL5 alone, although this did not seem to be relevant to kidney dysfunction. White bars, normal; black bars, edema; grey bars, adverse. C, sham-injected control; NI, non-injected control; n = 32–68 embryos/injection batch; masked scoring. (TIF) [file pgen.1005349.s001.tif]

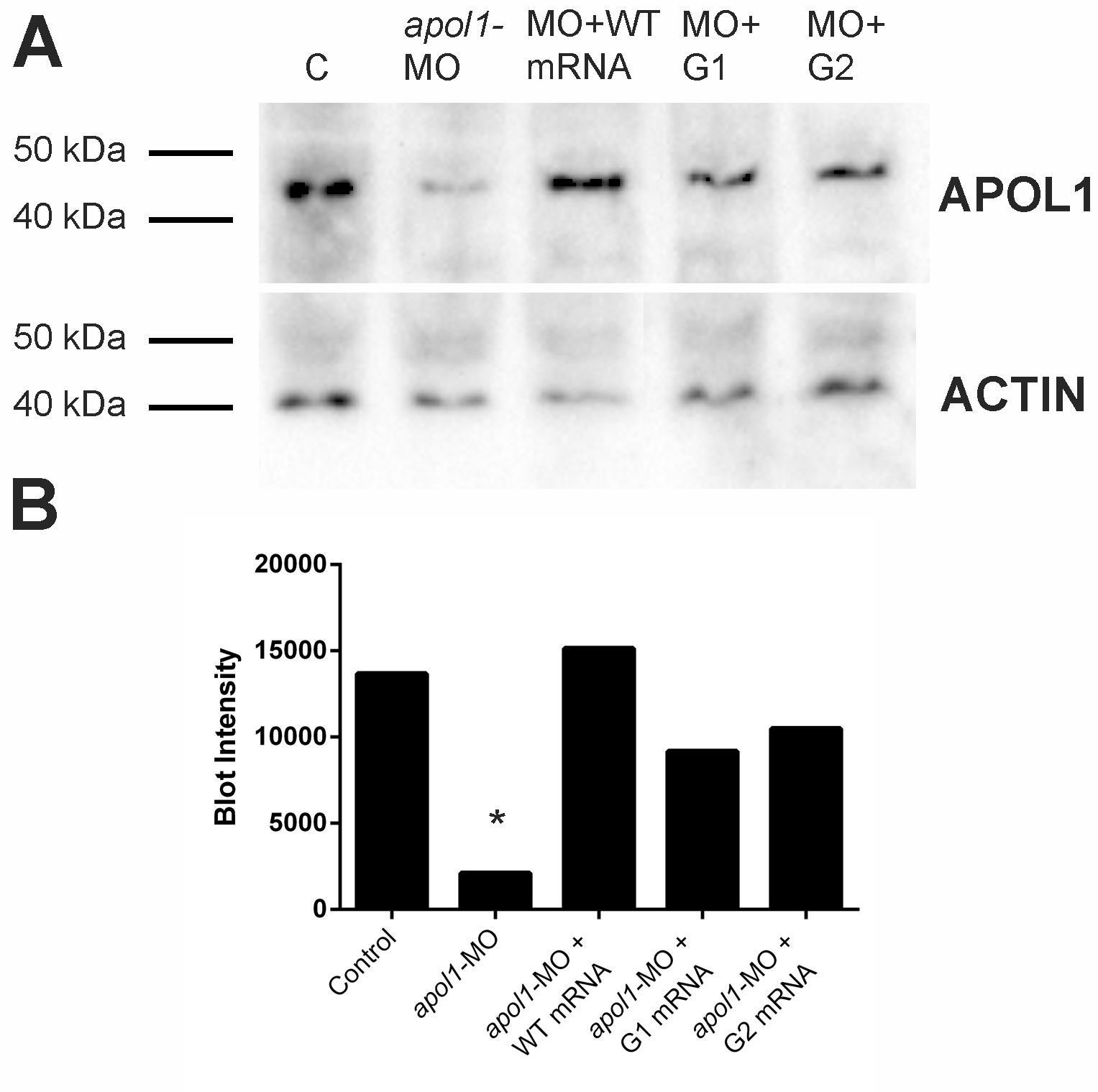

Supplement: S2 Fig — Protein lysates from zebrafish embryos injected with apol1-MO (1.0ng/nl) alone or co-injected with either wild-type, G1, or G2 APOL1 human mRNA (100pg) were isolated from 2 dpf embryos. (A) APOL1 protein levels were assessed by Western blot (Abcam EPR2907) and (B) pixel intensity normalized to ACTIN was calculated for comparison. (A-B) Embryos injected with translation-blocking apol1-MO display a significant reduction in APOL1 protein expression compared to non-injected controls, suggesting cross-reactivity with zebrafish APOL1 and efficiency of the apol1 MO to block translation. Protein levels are restored to control levels upon co-injection of wild-type, G1, or G2 APOL1 human mRNA. Blot shown is a representation of four independent experiments. Lane 1, non-injected control; Lane 2, apol1-MO injected; Lane 3, apol1-MO + wild-type APOL1 human mRNA; Lane 4, apol1-MO + G1 APOL1 human mRNA; Lane 5, apol1-MO + G2 APOL1 human mRNA. *p = 0.026. (PNG) [file pgen.1005349.s002.png]

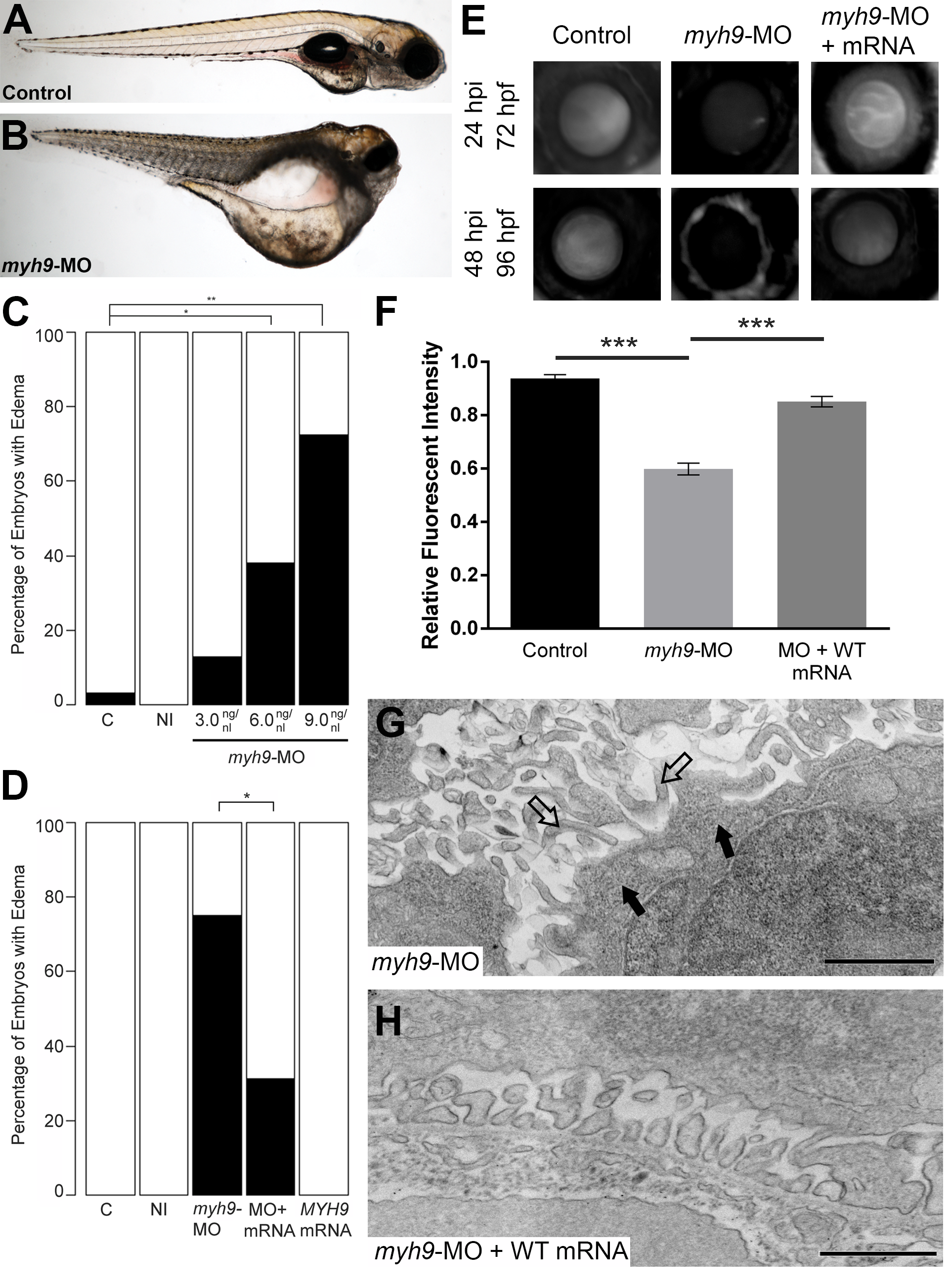

Supplement: S3 Fig — We recapitulated data reported by Müller et al. for experimental comparison [17]. (A-B) Representative live images of sham-injected control and myh9 morpholino (MO) injected larvae at 5 dpf. (C) Injection of increasing doses of myh9 MO demonstrate dose-dependent effects when scored for generalized edema compared to control embryos at 5 dpf. (E-F) myh9 morphants also display filtration defects indicated by significantly increased dextran clearance. (D-F) Co-injection of wild-type human MYH9 mRNA (100pg/nl) significantly rescues edema formation and filtration defects observed in myh9 morphants. (G) As reported previously by Müller et al., myh9 morphants display ultrastructure abnormalities, including glomerular basement membrane thickening and the presence of microvillus protrusions in the urinary space. (H) These ultrastructural defects are rescued upon co-injection of wild-type human MYH9 mRNA (100pg). White bars, normal; black bars, edema; n = 49–70 and n = 13–29 embryos/injection batch for gross morphological scoring and glomerular filtration assays, respectively; *p<0.05; **p<0.01; ***p<0.001; filled arrowheads, glomerular basement membrane; open arrowheads, microvillus protrusions. (TIF) [file pgen.1005349.s003.tif]

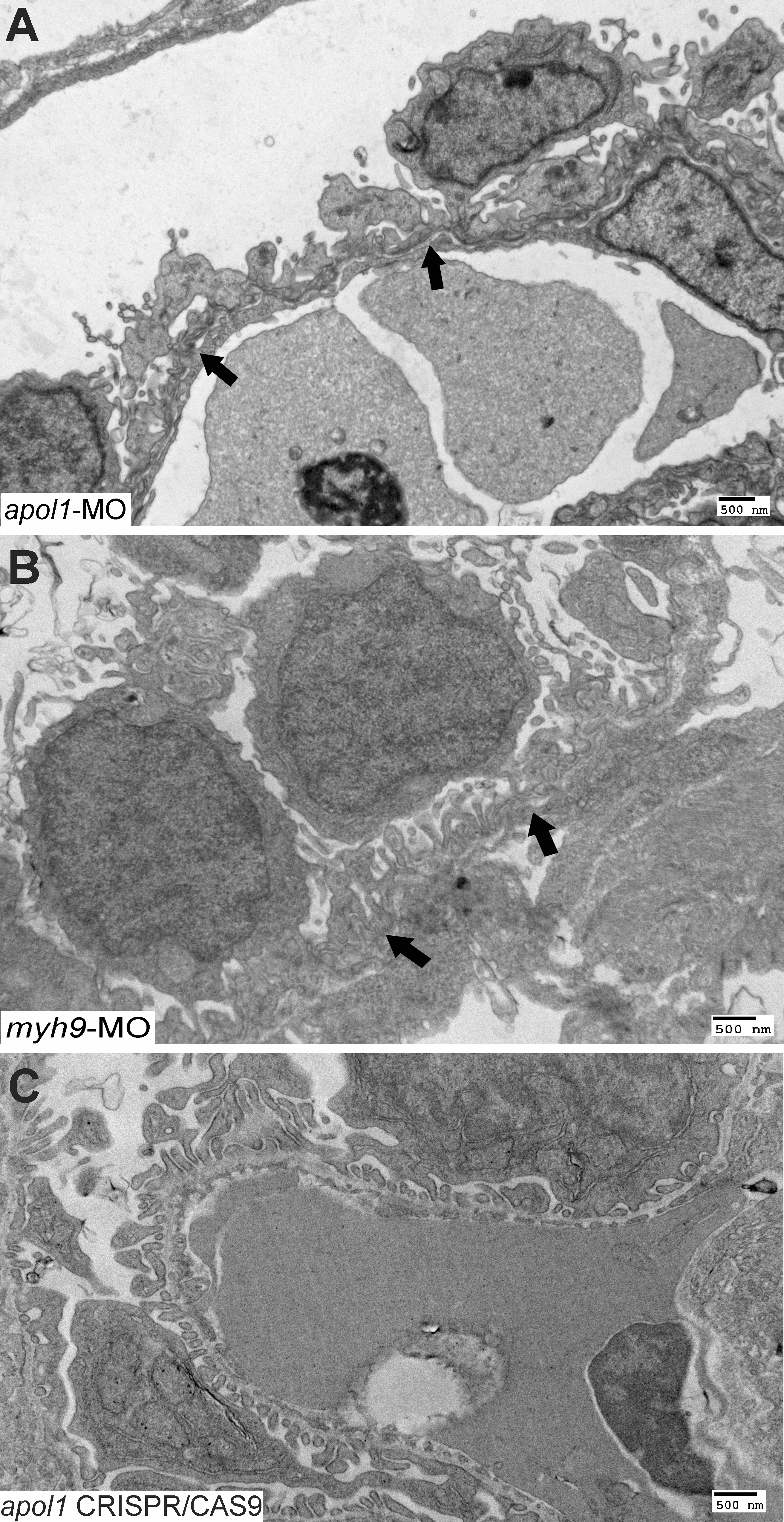

Supplement: S4 Fig — Transmission electron microscopy of zebrafish larval glomeruli injected with either (A) apol1-MO or (B) myh9-MO were imaged at 5 dpf using a low magnification (direct mag = 4400X) to characterize long stretches of the glomerular basement membrane (GBM). Comparatively, apol1 and myh9 morphants display similar abnormalities, including podocyte disorganization and effacement, as well as the presence of microvillus protrusions. However, myh9 morphants display a thickened GBM that is not apparent in apol1-MO injected larvae, while apol1 morphants appear to have a higher degree of podocyte effacement compared to myh9 morphants. (C) Zebrafish larvae injected with apol1 CRISPR/CAS9 display a similar glomerular ultrastructure compared to apol1 morphants at 5 dpf. Filled arrowheads, glomerular basement membrane. Scale bar = 500nm. (TIF) [file pgen.1005349.s004.tif]

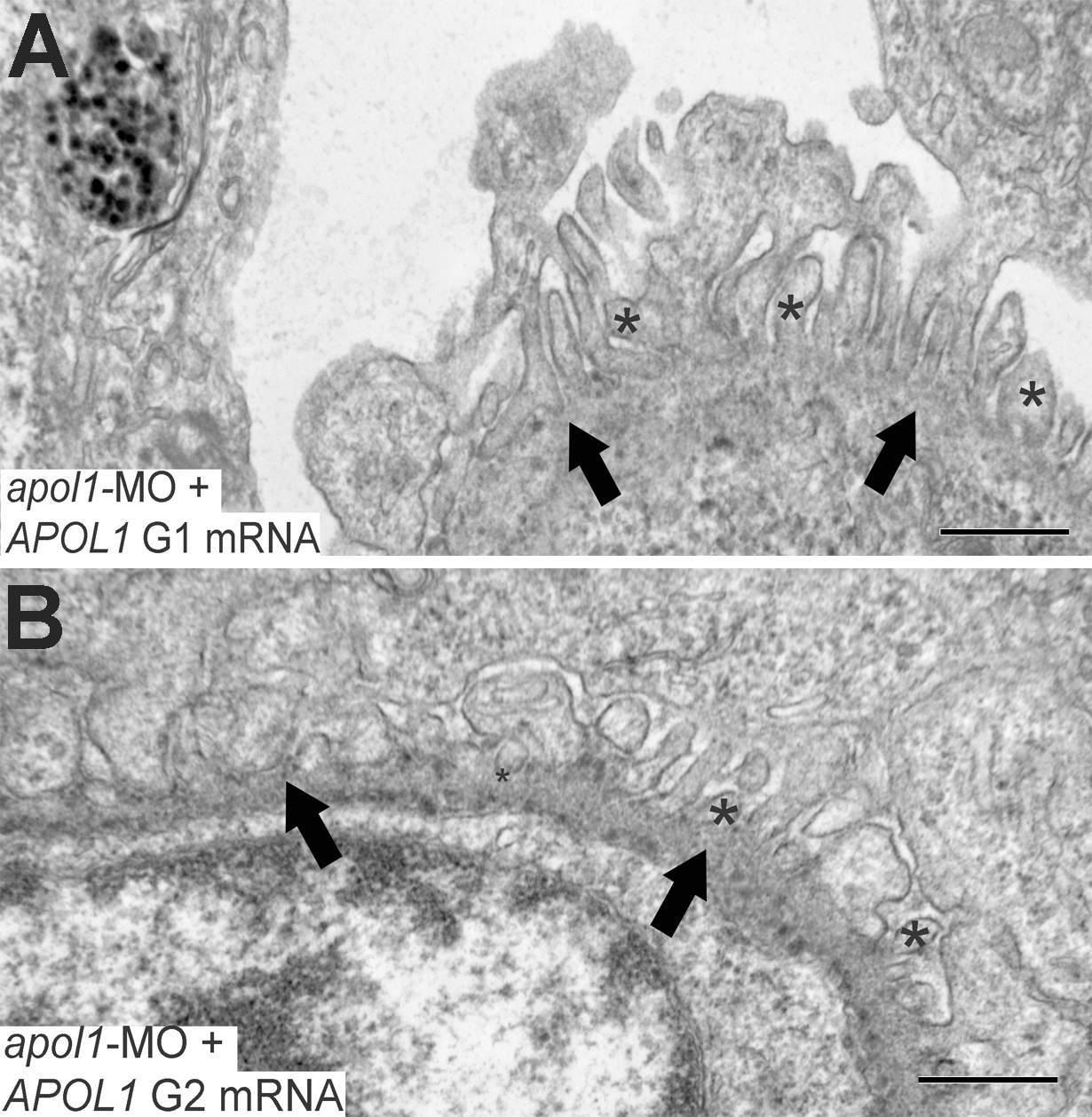

Supplement: S5 Fig — Transmission electron microscopy of zebrafish larval glomeruli imaged at 5 dpf. (A, B) apol1 morphants complemented with risk alleles, G1 and G2 do not rescue the observed defects caused by apol1 suppression, with naked patches of glomerular basement membrane and microvillus processes apparent. *, microvillus protrusions; filled arrowheads, glomerular basement membrane. Scale bars, 500nm. (TIF) [file pgen.1005349.s005.tif]

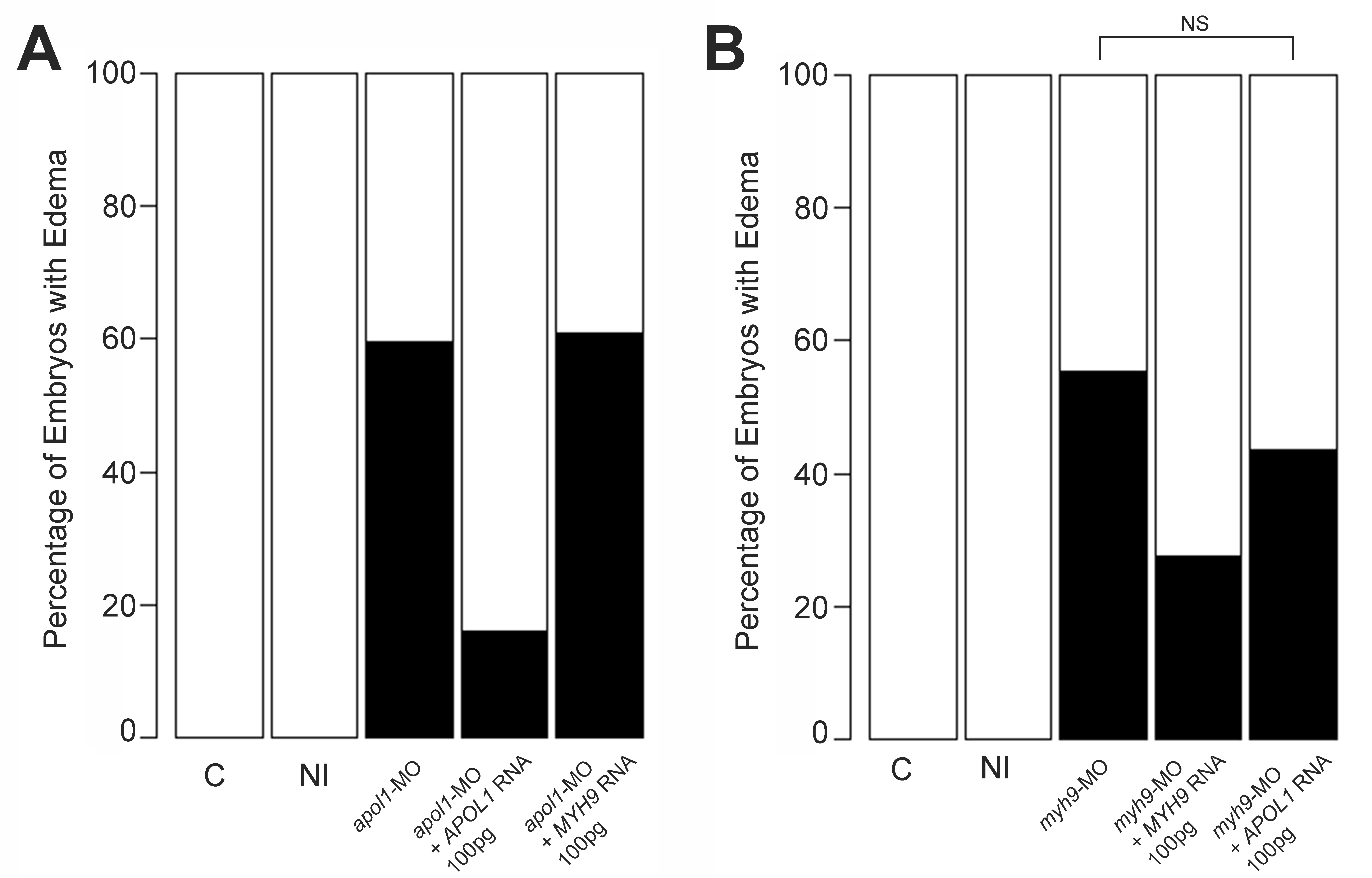

Supplement: S6 Fig — (A) apol1-MO was co-injected with human WT MYH9 mRNA (100pg/nl) and (B) myh9-MO was co-injected with human WT APOL1 mRNA; embryos were scored for edema formation at 5 dpf (n = 25–66 embryos/injection for apol1-MO/MYH9 RNA and n = 32–46 embryos/injection for myh9-MO/APOL1 RNA); each repeated three times. (TIF) [file pgen.1005349.s006.tif]

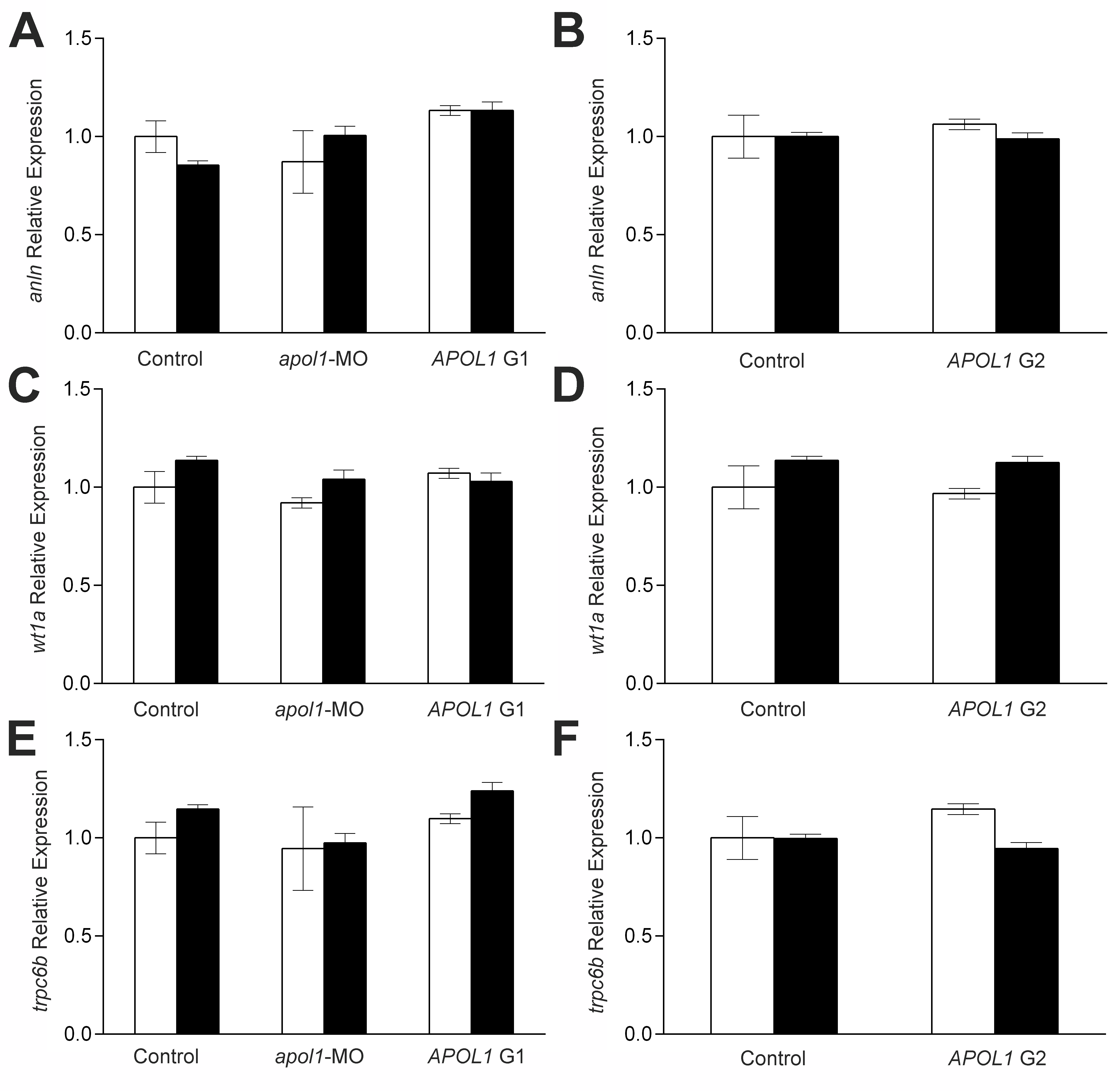

Supplement: S7 Fig — Zebrafish embryos were injected with either apol1-MO (1.0ng/nl dose), APOL1 G1 (S342G:I384M) mRNA (100pg), or APOL1 G2 (100pg) mRNA alone, in the absence (white bars) or presence (black bars) of atpif1α-MO. Total RNA at 5 dpf or 3 dpf (APOL1 G2/atpif1α-MO embryos did not survive to 5 dpf) was extracted and reverse-transcribed with random primers to obtain whole-embryo cDNA. (A-B) anln, (C-D), wt1a, (E-F) or trpc6b expression was determined by quantitative real-time PCR and relative expression was calculated against actb1. We observed no significant differences in expression in any of the FSGS-associated genes tested under apol1/APOL1 modulation, suggesting that APOL1 G2 regulation may be specific to myh9. White bars = normal; black bars = atpif1α-induced anemia. Relative expression values are mean ± SE in triplicate with two biological replicates. (TIF) [file pgen.1005349.s007.tif]
